# Genome architecture modulates 35S and 5S rDNA homogenization in Asteraceae

**DOI:** 10.1111/tpj.71174

**Published:** 2026-10-22

**Authors:** Daniel Vitales, Joan Pere Pascual‐Díaz, Veronika Zoulová, Ales Kovarik, Sònia Garcia

**Affiliations:** ^1^ Institut Botànic de Barcelona (IBB), CSIC‐CMCNB Barcelona Catalonia 08038 Spain; ^2^ Institute of Biophysics Academy of Sciences of the Czech Republic Brno 61265 Czech Republic

**Keywords:** ribosomal DNA, concerted evolution, selection, birth‐and‐death evolution, homogenization, genome architecture, linked arrangement, separate arrangement, copy number variants, single nucleotide polymorphism (SNP)

## Abstract

Concerted evolution is the model by which multicopy gene families are homogenized across genomes, with ribosomal DNA (rDNA) as its textbook example. However, the extent and efficiency of this process may vary depending on genomic factors. The plant family Asteraceae provides a natural comparative system to address the evolutionary dynamics of rDNA, as it is uniquely characterized by the coexistence of two contrasting genomic arrangements: a separate (S‐type) and a linked (L‐type) organization of 5S and 35S rRNA genes. Here we analyzed rDNA arrangements in 71 representative Asteraceae species (50 S‐type and 21 L‐type) and, using WGS data and repeatome analyses, we have reconstructed full rDNA units and quantified the intragenomic variability across coding (genic) and non‐coding (spacer) regions. No intermediate (coexisting L‐type+S‐type) genotypes were found. Our results reveal contrasting patterns of 5S rDNA variation depending on its genomic context. S‐type species exhibit high levels of 5S polymorphism in both genic and spacer regions, while L‐type species show near‐complete sequence homogeneity, consistent with variation in homogenization efficiency and selective constraints related to copy number and chromosomal position of 5S rRNA genes. In contrast, 35S rDNA shows a low polymorphism level overall. The findings support a context‐dependent view of concerted evolution across rDNA and highlight the influence of genome architecture on rDNA evolution. In particular, the contrasting behavior of 5S (highly conserved when linked but highly variable when separate) emerges as a key example of how structural genomic context shapes the evolution of even the most conserved housekeeping genes.

## Significance Statement

Ribosomal DNA is a classic model of concerted evolution, yet how genome organization influences its homogenization remains unclear. By comparing alternative rDNA arrangements across family Asteraceae, we show that genomic context strongly shapes intragenomic variation, revealing that even highly conserved gene families can follow contrasting evolutionary trajectories depending on their chromosomal organization.

## INTRODUCTION

Nuclear ribosomal DNAs (rDNAs) are key actors of all genomes. These ubiquitous genes encode ribosomal RNAs, fundamental components of ribosomes that facilitate the translation of messenger RNA (mRNA) into proteins, thus being critical to cell function and growth. Ribosomal RNAs account for ca. 80% of cellular RNAs (Antony et al., 2022) despite rDNAs only making up a small fraction of the genome, typically well below 1% (e.g., 0.13% in bread wheat; Tulpová et al., 2022) but in small genomes such as Arabidopsis, it can reach 6–8% (Barakat et al., 1998). Beyond their essential role in protein synthesis, rDNAs contribute to genomic stability and are related to DNA damage response or longevity (Ganley & Kobayashi, 2014; Garcia et al., 2024; Kobayashi, 2006).

The repetitiveness is a defining feature of ribosomal DNA, impacting its genome organization, functions, and evolutionary dynamics. There are hundreds to thousands of rDNA repeats per genome, typically tandemly arranged. Such high copy numbers reflect the high cellular demand for ribosomes (McStay & Grummt, 2008; Welfer et al., 2025) and secure redundancy, so cells can continue producing ribosomes even if there is epigenetic silencing or damage of any rDNA unit. The sequence integrity of rDNA is maintained through the mechanism known as concerted evolution, a process by which the copies of repeated sequences evolve coordinately, and thus a high similarity is maintained across copies and loci (Brown et al., 1972; Zimmer et al., 1980). Concerted evolution is not unique to rDNA but applies to other multigene families such as tandemly‐arranged histone genes (Nei & Rooney, 2005). However, rDNA is considered its best‐known example. The main molecular mechanisms underlying this process include unequal crossing over and gene conversion (Dover, 1982), which together act to homogenize tandem arrays while altering variant frequencies. Finally, variants containing mutations within rRNA genes would generally be selected against, while chromosomes containing mutations in the non‐coding regions of the unit would be under no adverse selective pressure (Eickbush & Eickbush, 2007). By these processes, rDNA arrays typically maintain high sequence similarity over time. However, the contribution of purifying selection to the concerted evolution of rDNA has rarely been discussed, although recent work by Sultanov and Hochwagen (2022) argues for its central role.

Ribosomal DNAs are organized as tandemly repeated units located at one or more chromosomal loci (Figure 1). In eukaryotes, the genic regions of rDNA repeat comprise the 18S, 5.8S, and 25S/28S rRNA genes (referred to as 45S in animals and 35S in plants), which are transcribed—together with the internal transcribed spacers named ITS1 and ITS2—as a single precursor by RNA polymerase I (Hemleben & Zentgraf, 1994; Volkov et al., 2022). Each repeat unit of coding regions is separated from the next by a long intergenic spacer (IGS), including the external transcribed spacer (ETS), a non‐coding region of the rDNA transcription unit that is found upstream of the 18S rRNA gene, and sometimes also downstream of the 25S/28S rRNA gene. When transcriptionally active, these 35S/45S arrays form the nucleolar organizer regions (NORs) (Long & Dawid, 1980). Separately, the small 5S rRNA gene (typically around 120 bp), transcribed by RNA pol III (White, 2011), is also arranged in tandem arrays, with each copy separated by a non‐transcribed spacer (NTS). This is the canonical rDNA organization in most eukaryotes, including plants, termed separate arrangement of rRNA genes (S‐type) (Figure 1a–c). Yet there is a much less usual arrangement in which the 5S rRNA gene repeats are physically linked to the 45S/35S rDNAs, embedded in the IGS (called L‐type) (Figure 1d–f). The broad but scattered distribution of the rDNA linkage suggests that this arrangement has been maintained and repeatedly gained or lost during evolution, implying distinct evolutionary pressures and trade‐offs (see reviews by Wicke et al., 2011, Garcia et al., 2024). It has been found in fungi, protists, and a few groups of animals and plants (Drouin & de Sa, 1995). Among animals, the vast majority have 5S rDNA on chromosomes outside 45S, and only certain turtles, crocodilians, and Antarctic fish show 5S integrated into the 45S array (Dyomin et al., 2023). In seed plants there is a similar scenario, and separate 5S arrays are also typical, although linked 35S‐5S units have emerged in gymnosperms families such as Ginkgoaceae, Ephedraceae, or Podocarpaceae (Galián et al., 2012; Garcia & Kovařík, 2013) and in several groups of the large angiosperm family Asteraceae (Garcia et al., 2009, 2010, 2012).

**Figure 1:**
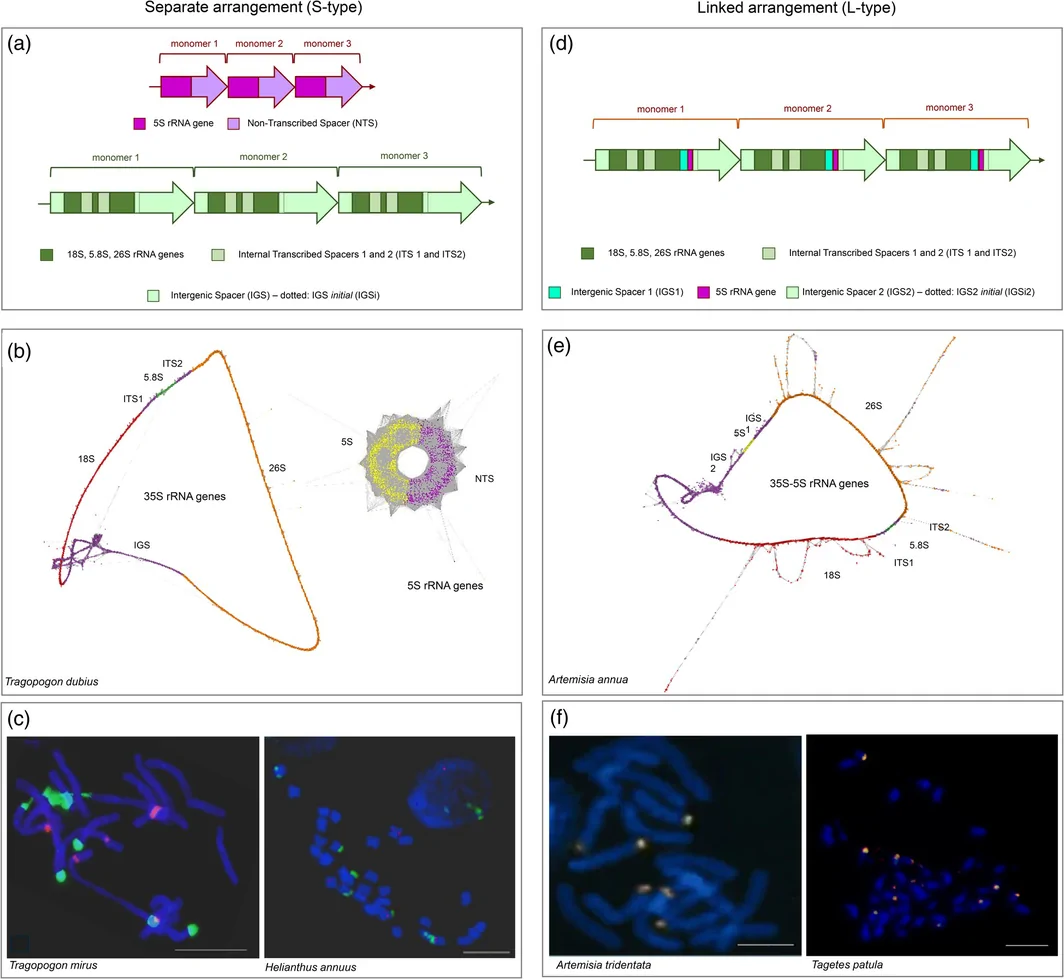
Separate (S‐type) versus linked (L‐type) organization of 35S and 5S rDNA units. (a) Schematic representation of the separate arrangement (S‐type), in which tandem arrays of 5S rDNA (5S rRNA gene + non‐transcribed spacer, NTS) and 35S rDNA (18S–ITS1–5.8S–ITS2–26S + intergenic spacer, IGS) occur as independent repeats (monomers). (b) Representative RepeatExplorer graph‐based reconstruction for an S‐type species (*Tragopogon dubius*), showing the main rDNA clusters: a 35S rDNA path (with annotated 18S, ITS1, 5.8S, ITS2, 26S and IGS regions) and a separate 5S rDNA cluster constituted by the 5S gene and NTS. (c) Dual‐color FISH examples consistent with the S‐type arrangement (left: *Tragopogon mirus*; right: *Helianthus annuus*), showing 35S and 5S rDNA signals at distinct chromosomal/nuclear sites (reprinted from Garcia et al., 2010, with permission). (d) Schematic representation of the linked arrangement (L‐type), where 35S and 5S rDNA are physically linked within the same tandemly repeated unit (35S–IGS1–5S–IGS2), defining a single composite monomer. (e) Representative RepeatExplorer graph‐based reconstruction for an L‐type species (*Artemisia annua*), showing a single linked cluster that contains both 35S and 5S regions within the same repeat, separated by two IGS segments (IGS1 and IGS2). (f) Dual‐color FISH examples consistent with the L‐type arrangement (left: *Artemisia tridentata*; right: *Tagetes patula*), showing predominantly co‐localized or closely overlapping 35S and 5S signals (overlap visible as yellow‐orange). In (c, f), chromosomes/nuclei are counterstained with DAPI (blue); 35S and 5S rDNA signals are shown in green and red, respectively (overlap = orange). Scale bars as indicated. Reproduced from Garcia et al. (2009) Chromosoma 118:85–97 and from Garcia et al. (2010) BMC Plant Biology 10:176, licensed under CC BY 2.0.

The family Asteraceae is one of the largest and most diverse of angiosperms, with ca. 25 000–35 000 species, accounting for c. 10% of all flowering plants (Mandel et al., 2019). With an amazing diversity in morphology, ecological ranges, and worldwide distribution, Asteraceae occur in most ecosystems, from steppes to rainforests, and have been detected even in Antarctica (Lewis Smith & Richardson, 2011). The evolutionary success of this family is attributed to its high adaptability and complex reproductive strategies, particularly for its unique composite flower head, the capitulum, which groups many small individual florets into a single structure that attracts pollinators efficiently (Elomaa et al., 2018). Asteraceae stands out as an exceptional model for the study of ribosomal DNA evolution, as the unique coexistence of both L‐type and S‐type rDNA organizations, in some cases even in closely related species (Garcia et al., 2009), provides a rare opportunity to investigate the evolutionary implications of these different rDNA genomic configurations. Besides, the existence of species with varying genome sizes and ploidy levels in the family provides an ideal framework to explore how genome complexity influences rDNA evolution.

Here, we investigate patterns of rDNA variation across 71 Asteraceae species representing both S‐type and L‐type arrangements. Specifically, we will (1) study the intragenomic variability of 5S and 35S rRNA genes, characterizing polymorphisms of genic and non‐genic regions in a phylogenetic context; (2) analyze the intragenomic variability concerning the type of polymorphism and its frequency, rDNA abundance (copy number), or locus number; and (3) compare levels of intragenomic variability in species having S‐type and L‐type rDNA arrangements. By using Asteraceae as a comparative system, we aim to assess how genome organization shapes rDNA evolution and to place the classical model of concerted evolution in a broader, context‐dependent framework.

## RESULTS

### Plastome and 35S rDNA phylogenetic analysis

To provide a phylogenetic framework in which to analyze rDNA evolution of these species, the sequences of tRNA, rRNA and CDS plastome regions for the 71 Asteraceae taxa (plus one outgroup of family Calyceraceae) in our dataset (Table S1) were concatenated in an alignment of 84 268 nucleotides (Data S1). The resulting phylogenetic trees obtained using Bayesian and maximum‐likelihood (ML) approaches are presented in Figure S1 and Figure S2, respectively. The topology of both trees is largely congruent and most nodes show strong statistical support (PP > 0.95; BS > 95%), with the exception of: (i) the placement of tribe Senecioneae relative to the remaining tribes of Asteroideae (PP = 0.58; BS = 57%); (ii) the split between tribes Gochnatieae and Hyalideae (PP = 0.81; BS < 50%); (iii) the split between *Calotis plumulifera* F.Muell. and *Iotasperma sessilifolium* (F.Muell.) G.L.Nesom (PP = 0.72; BS < 50%) within tribe Astereae. The first two poorly supported relationships at the backbone of our tree represent conflicting evolutionary relationships that have also been reported in previous phylogenetic studies (e.g. Mandel et al., 2019; Panero & Crozier, 2016; Pascual‐Díaz et al., 2021; Zhang et al., 2021). The ML reconstruction (Figure S2) showed lower resolution at several additional nodes, including the relationship between tribes Anthemideae and Astereae (BS = 85%), the relationships among Heliantheae, Eupatorieae, Millerieae, and Madieae (BS = 56%), and the placement of Mutisieae relative to the remaining derived tribes of the family (BS = 78%). Nevertheless, the phylogenetic positions of all tribes represented in our dataset were consistent with those recovered in recent phylogenetic reconstructions of the family based on nuclear (Mandel et al., 2019; Zhang et al., 2021) and plastome data (Panero & Crozier, 2016; Pascual‐Díaz et al., 2021). All the taxa in our dataset are also placed within the tribe formerly assigned to them according to previous systematic classification (POWO, 2025).

The phylogenetic reconstructions of 35S rDNA evolution produced trees with strong support (PP > 0.95; BS > 95%) for most terminal nodes in both Bayesian and ML analyses (Figures S3 and S4, respectively). The relationships inferred among species within each tribe were largely congruent with those recovered in the plastome‐based phylogeny. However, several relationships at the backbone of the trees showed low resolution, particularly those among the main Asteroideae tribes, which formed an unresolved polytomy in both the Bayesian and ML trees. Overall, the Bayesian and ML topologies were largely congruent, although the ML tree showed lower support for some deeper relationships (Figure S4). Therefore, given the limited resolution of the 35S rDNA phylogeny at deeper taxonomic levels, and because the Bayesian plastome phylogeny provided a well‐resolved topology that was congruent with previous nuclear‐based phylogenetic studies, subsequent phylogenetic signal analyses were based on the Bayesian plastome phylogeny.

### Ribosomal DNA reconstruction

The 35S and 5S rDNA units (as well as the linked 35S‐5S rDNA units in the case of L‐type species) from all the samples of this study were fully reconstructed (excluding IGS regions) with the TAREAN bioinformatic pipeline. The circular graph clusters obtained in all cases indicate the successful reconstruction of complete rDNA units, enabling the generation of a consensus sequence for each (Figure 1). Out of the 71 species of Asteraceae here analyzed (Table S1), 50 showed S‐type arrangement, evidenced by two separate TAREAN graph clusters, each of them containing sequences annotated either as 35S or as 5S genic regions. Exceptionally, for *Tragopogon dubius* Scop. (Cichorieae), the reconstruction resulted in separate 35S and 5S clusters, as well as an additional smaller cluster also annotated as 5S rDNA. The remaining 21 species (representing tribes Anthemideae, Gnaphalieae, Coreopsidae and Tageteae) showed L‐type configuration of rDNA, evidenced by the presence of reads annotated as 35S and 5S genic regions in the same graph cluster.

The rDNA reconstruction of *Matricaria chamomilla* L. (Anthemideae) resulted in one cluster with 35S and 5S linked in the same unit, as well as an independent smaller cluster only containing 5S annotated reads which are consistent with Cassandra TRIM sequences. In addition, we validated the rDNA organization inferred by TAREAN by Southern blot hybridisation using 5S‐ and 26S‐derived probes on BamHI‐digested genomic DNA from a subset of species predicted to have linked (*Artemisia annua* L., *A. tridentata* Nutt., *M. chamomilla, Chamaemelum nobile* L., *Helichrysum splendidum* (Thunb.) Less., *Tagetes patula* L., *Coreopsis gigantea* (Kellogg) H.M.Hall) and unlinked arrays (*Conyza bonariensis* L., *Dittrichia viscosa* (L.) Greuter, *Arnica montana* L.). Because BamHI cleaves DNA at a defined recognition site, it generates a reproducible set of restriction fragments: when 5S and 26S rDNA are physically linked, digestion can produce fragments containing both loci and the same band is detected by both probes, whereas unlinked arrangements yield distinct bands for each probe (Figure S5).

The 35S rDNA units contain the 18S‐5.8S‐26S coding regions, as well as the internal transcribed spacers (ITS1 and ITS2) and a long intergenic spacer (IGS) showing numerous subrepeats clearly depicted in the mapping analysis. The total length of the recovered 18S‐5.8S‐26S regions (including ITS1 and ITS2 sequences and excluding the IGS) in L‐type and S‐type species varied from 5825 bp in *C. plumulifera* (S‐type) to 5856 bp in *Stevia rebaudiana* Bertoni (L‐type) (Table S2). All of the reconstructed 5S rDNA units from S‐type species share a conserved 120 bp genic region while they differ in the length of the intergenic spacer NTS (122–410 bp; see Table S2). In species with linked 35S‐5S rDNA units, apart from the 18S‐5.8S‐26S coding regions (length ranging between 5821 and 5851 bp; see Table S2), the intergenic region between 35S and 5S genes (named IGS1 in these species) shows considerable length variation (from 131 to 1124 bp) while the 5S rDNA genic region also presents the typical 120 bp conserved length. The long intergenic spacer of S‐type and L‐type species (named IGS and IGS2, respectively) contains numerous subrepeats that vary in number among the copies of the unit, preventing their estimated size from being employed for subsequent comparative analyses.

### Intragenomic variability in genic and non‐genic regions of 35S and 5S rRNA genes

The average intragenomic variability (expressed in number of polymorphisms per 100 bp, taking into account all type of nucleotide and structural polymorphisms) among rDNA regions of the 71 Asteraceae species analyzed in this study is summarized in Table 1 (see Table S3 for absolute polymorphism values in each species). The average intragenomic variability values (considering all polymorphism types) within all regions analyzed range between 0.15 (in 18S gene) and 5.99 (in 5S's NTS). The genic regions of 35S rDNA show low intragenomic variability (Figure 2), with similar values among 18S, 5.8S and 26S genes (ranging 0.15–0.22 polymorphisms per 100 bp). The two intergenic spacers (ITS1 and ITS2, hereafter referred to as “ITS” unless otherwise indicated) show very similar average polymorphism values (around 1.1 polymorphisms per 100 bp), but their intragenomic variability levels are 5.75‐fold higher than in the 35S genic regions. The 5S gene, despite its reduced size, shows around seven times higher variability than the average value of all 35S genic regions (1.3 versus 0.19 polymorphisms per 100 bp, respectively) (Figure 2).

**Table 1:** Average intragenomic variability (polymorphisms per 100 bp) among rDNA regions of 71 Asteraceae species. The intragenomic variability of rDNA regions is differentiated among (i) nucleotide (single nucleotide variant and multiple nucleotide variant) and structural (insertion, deletion and replacement) polymorphisms and (ii) frequency of intragenomic polymorphisms (high frequency if present in more than 10% of the copies or low frequency if present in less than 10% of the copies). Absolute variability values per species are presented in Table S5

	18S	ITS1	5.8S	ITS2	26S	NTS	5S	IGS1	IGS2i	IGSi
High frequency	0.08	0.95	0.14	0.92	0.16	4.17	0.50	2.27	1.41	1.36
% high freq	57.1	85.8	64.0	80.3	74.0	69.6	37.2	75.8	76.6	89.9
Low frequency	0.06	0.16	0.08	0.23	0.06	1.82	0.84	0.73	0.43	0.15
% low freq	42.9	14.2	36.0	19.7	26.0	30.4	62.8	24.2	23.4	10.1
Nucleotide	0.14	1.06	0.21	1.08	0.22	5.15	1.07	2.08	1.52	1.07
% nucleotide	95.2	95.6	100.0	94.7	97.7	86.1	79.9	69.6	83.0	71.4
Structural	0.01	0.05	0.00	0.06	0.00	0.83	0.27	0.91	0.31	0.43
% structural	4.8	4.4	0.0	5.3	2.3	13.9	20.1	30.4	17.0	28.6
Total	0.15	1.11	0.21	1.14	0.22	5.99	1.34	3.00	1.83	1.51

**Figure 2:**
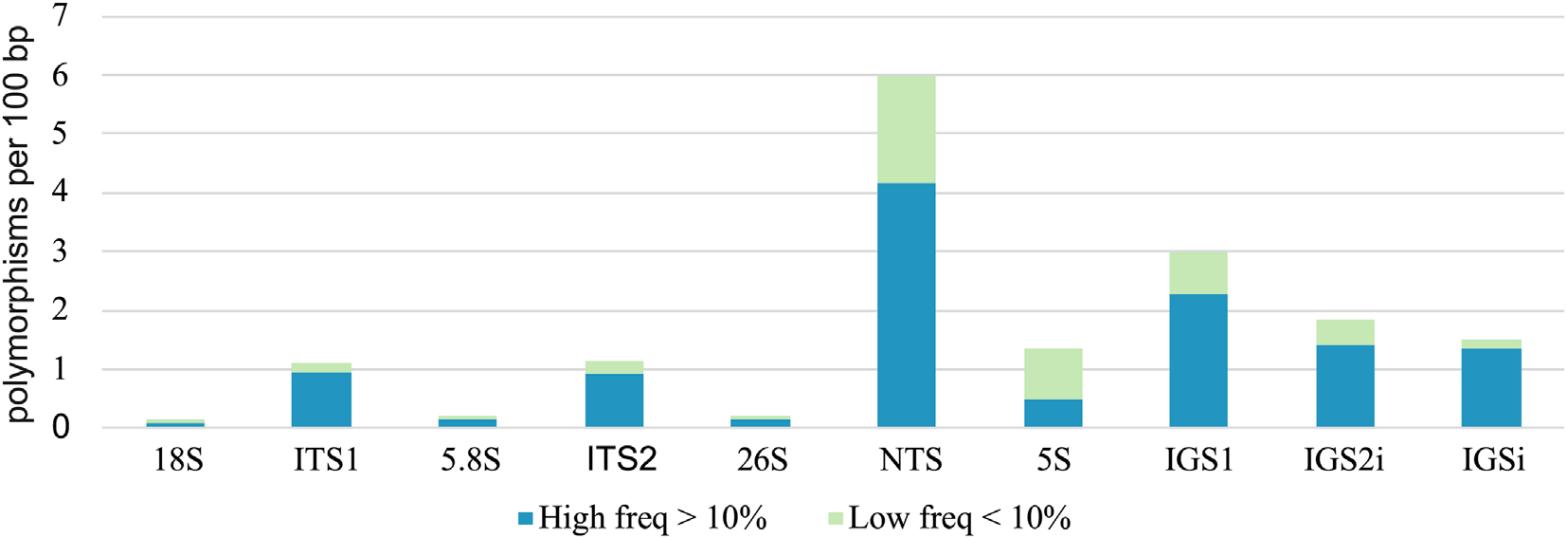
Average number of high frequency and low frequency polymorphisms per species across 5S and 35S rDNA regions. Bars represent the mean values across species for each rDNA region. Dark green indicates high‐frequency polymorphisms, present in >10% of rDNA copies, whereas light green indicates low‐frequency polymorphisms, present in <10% of copies.

These differences were strongly associated with rDNA organization (Table 2). Average 5S variability was markedly higher in S‐type species than in L‐type species (1.87 versus 0.08 polymorphisms per 100 bp, respectively) (Figure 3), and this difference was highly significant (Kruskal‐Wallis χ^2^ = 15.55; *P* < 0.0001). Nearly all L‐type species lacked 5S polymorphisms, with only *Achillea millefolium* L. and *Rhodanthe collina* Paul G.Wilson showing detectable variation. In contrast, 35S variability was slightly higher in L‐type than in S‐type species (0.32 versus 0.14 polymorphisms per 100 bp) (Figure 3), although this difference was not significant (Kruskal‐Wallis χ^2^ = 0.43; *P* = 0.5115; Table 3). The ITS region also showed higher intragenomic variability in L‐type than in S‐type species (1.57 versus 0.94 polymorphisms per 100 bp), in this case with significant differences among both groups (Kruskal‐Wallis χ^2^ = 4.55; *P* = 0.0328).

**Table 2:** Average and range of intragenomic variability (polymorphisms per 100 bp) among rDNA regions of S‐type and L‐type Asteraceae species

	S‐type species		L‐type species	
	Range	Average	Range	Average
35S genic	0–1.62	0.14	0–2.82	0.32
5S genic	0–13.33	1.87	0–0.83	0.08
ITS	0–6.03	0.94	0–12.58	1.57
NTS	0–16.67	5.99	n/a	n/a
IGSi	0–8.40	1.51	n/a	n/a
IGS1	n/a	n/a	0–7.74	3.00
IGS2i	n/a	n/a	0–8.20	1.83

**Figure 3:**
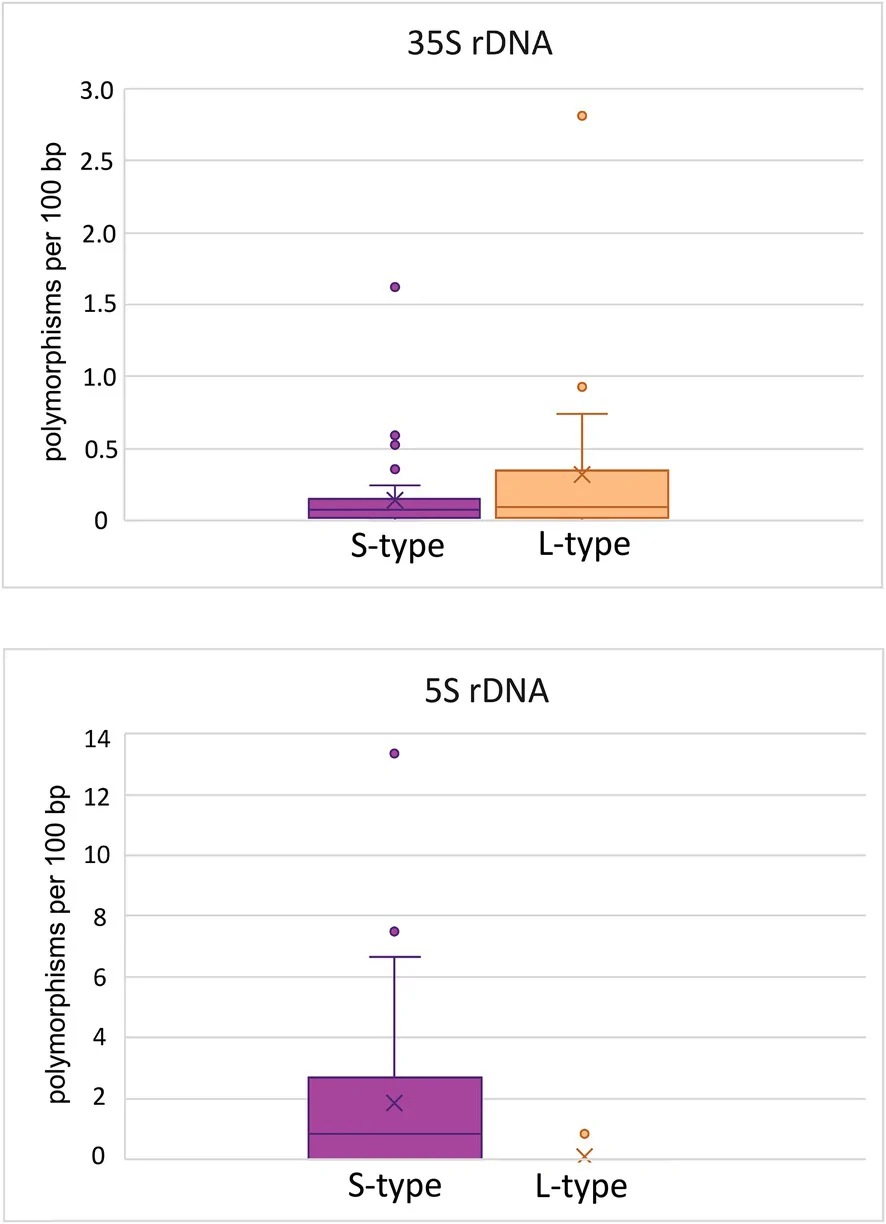
Boxplots of intragenomic polymorphisms in 35S (upper panel) and 5S (lower panel) rDNA regions among species showing separated arrangement (S‐type; purple) and species showing the linked arrangement (L‐type; orange). Polymorphism density is expressed as the number of polymorphisms per 100 bp. Boxplots show the median (horizontal line), interquartile range (box) and mean (cross); points indicate outliers.

**Table 3:** Summary of statistical tests assessing associations between average intragenomic polymorphisms and (i) rDNA configuration (L/S‐types) (ii) rDNA genomic proportion, (iii) rDNA copy number and (iv) rDNA FISH signals (2n) across Asteraceae species in both a phylogeny‐dependent (Phylogenetic Generalized Least Squares; PGLS) and phylogeny‐independent (Kruskal‐Wallis test or Spearman correlation) context.

	*N*	35S		ITS1‐ITS2		5S	
		rho/χ^2^	*P*‐value	rho/χ^2^	*P*‐value	rho/χ^2^	*P*‐value
rDNA configuration (L/S)							
Kruskal‐Wallis	71	0.4301	0.5115	**4.5552**	**0.0328**	**15.552**	**<0.0001**
PGLS		–			**0.0052**		**0.0172**
Genomic proportion							
Spearman correlation	71	0.2237	0.0607	0.1930	0.1069	**0.4881**	**<0.0001**
PGLS			**–**		**0.0001**		**<0.0001**
Copy number							
Spearman correlation	44	0.1818	0.2375	0.2394	0.1176	**0.4467**	**0.0024**
PGLS			**–**		**<0.0001**		**<0.0001**
FISH signals							
Spearman correlation	17	**0.5451**	**0.0236**	**0.6448**	**0.0052**	**−0.546**	**0.0231**

*Note:*  Results with *P* < 0.05 were considered statistically significant and are indicated in bold.

The non‐transcribed spacer (NTS) of 5S is the region showing the highest variability (5.99 polymorphisms per 100 bp), followed by the intergenic regions of 35S‐5S rDNA in L‐type species (IGS1, showing 3.00 polymorphisms per 100 bp). The initial, subrepeat‐free parts of IGS and IGS2 regions (named IGSi and IGS2i in our study, see Material and Methods section and Figure 1 for further explanations) show similar variability values (1.51 and 1.83 polymorphisms per 100 bp in IGSi and IGS2i, respectively). A significant positive correlation between polymorphism values of 35S genic regions and ITS regions (*P* <0.0001; rho = 0.6419) in each species, as well as between polymorphism values of 5S and NTS (*P* = 0.0009; rho = 0.4557), are found. In contrast, no correlation is detected between polymorphism values of 35S and 5S genic regions, either considering the whole dataset (*P* = 0.7792) or separating S‐type (*P* = 0.9415) and L‐type species (*P* = 0.0512).

On average, nucleotide polymorphisms (SNV and MNV) are more abundant than structural polymorphisms (insertions, deletions and replacements) in all the analyzed rDNA regions. Nucleotide polymorphisms range from 69.6% (in IGS1) to 100% (in 5.8S) of total intragenomic variants (Table 1). In general, structural polymorphisms show higher proportions in 5S than in 35S regions (20.1 and 13.9% in 5S and NTS, respectively; 0 to 5.3% in 35S regions, including ITS1 and ITS2). IGS1 (in L‐type species) and IGSi (in S‐type species) are the regions showing higher proportions of structural variability (around 30% of total polymorphisms).

Based on the frequency of intragenomic polymorphisms, we classified polymorphisms as “high frequency” if they were present in more than 10% of the copies or “low frequency” if they were present in 5–10% of the copies. High‐frequency polymorphisms are generally more abundant than low‐frequency polymorphisms in our dataset (Figure 2). Particularly, high‐frequency polymorphisms are more common in intergenic (around 80% of polymorphic sites, on average) than in genic regions (around 60% of polymorphic sites) (Table 1). The 5S genic region represents a notable exception to the general pattern, being the only region showing considerably more low‐frequency polymorphisms (62.8%) than high‐frequency polymorphisms (37.2%). The difference in polymorphism abundance between coding and non‐coding regions is smaller for low‐frequency polymorphisms (approximately 3–4 fold) than for high‐frequency polymorphisms (around 10 fold) in both the 35S and 5S regions (Table 1). In other words, low‐frequency polymorphisms are more similarly distributed in coding and non‐coding regions, while high‐frequency polymorphisms are much more abundant in non‐coding than in coding regions.

### Phylogenetic signal of rDNA intragenomic variability and organization

The analysis of the phylogenetic signal of rDNA variability revealed contrasting evolutionary patterns across rDNA regions. The intergenic region ITS showed a very strong and highly significant phylogenetic signal (λ = 1.00, *P* <0.001), indicating that its degree of intragenomic variability closely mirrors the phylogenetic relationships within Asteraceae. In contrast, the variability of the 35S rDNA genic region lacked any detectable phylogenetic signal (λ ≈ 0, *P* = 1), suggesting it is independent of phylogenetic relatedness. The 5S rDNA region showed a low to moderate phylogenetic signal (λ = 0.31), which was not statistically significant (*P* = 0.10), indicating a weak tendency for related species to share similar levels of 5S rDNA variability.

Given the contrasting phylogenetic signal among rDNA regions, we next tested whether intragenomic variability differed between L‐ and S‐type species while accounting for phylogenetic relationships (Table 3). Intragenomic variability of ITS differed significantly between the two rDNA configurations after accounting for phylogenetic relationships (PGLS, *P* = 0.0052), with higher polymorphism in L‐type species. In contrast, intragenomic variability of 5S was significantly higher in S‐type than in L‐type species (PGLS, *P* = 0.0172). The 35S genic region was not included in the PGLS analysis because no detectable phylogenetic signal was observed for this region (λ ≈ 0).

The ancestral character state reconstruction of 35S and 5S rDNA organization (L‐type or S‐type) shows that the most recent common ancestor (MRCA) of Asteraceae, as well as the earliest‐diverging lineages of the family, are estimated to have a separate arrangement (S‐type) of rDNA (Figure 4). Only the MRCAs of each of the tribes Anthemideae, Gnaphalieae and Coreopsideae (i.e. tribes showing L‐type rDNA in all their species included in this dataset) are inferred to have an ancestral L‐type arrangement of rDNA. However, our results show that the ancestral character state of Tageteae, a tribe presenting both L‐type and S‐type species, is the latter. Regarding Asteroideae, the only subfamily within Asteraceae where linked arrangement has been detected so far, the MRCA is also inferred as S‐type. As for supertribe Asterodae, including tribes Senecioneae, Anthemideae, Astereae, Gnaphalieae and Calenduleae (this last tribe not being included in our dataset), the ancestral character state would also be S‐type. According to our results, the MRCA of the clade formed by Anthemideae, Astereae and Gnaphalieae likely had a S‐type rDNA (62% probability). The same pattern is found for the ancestral character state of the clade constituted by Anthemideae and Astereae, where the stochastic character mapping detects a high level of uncertainty (62% probability of S‐type ancestral state).

**Figure 4:**
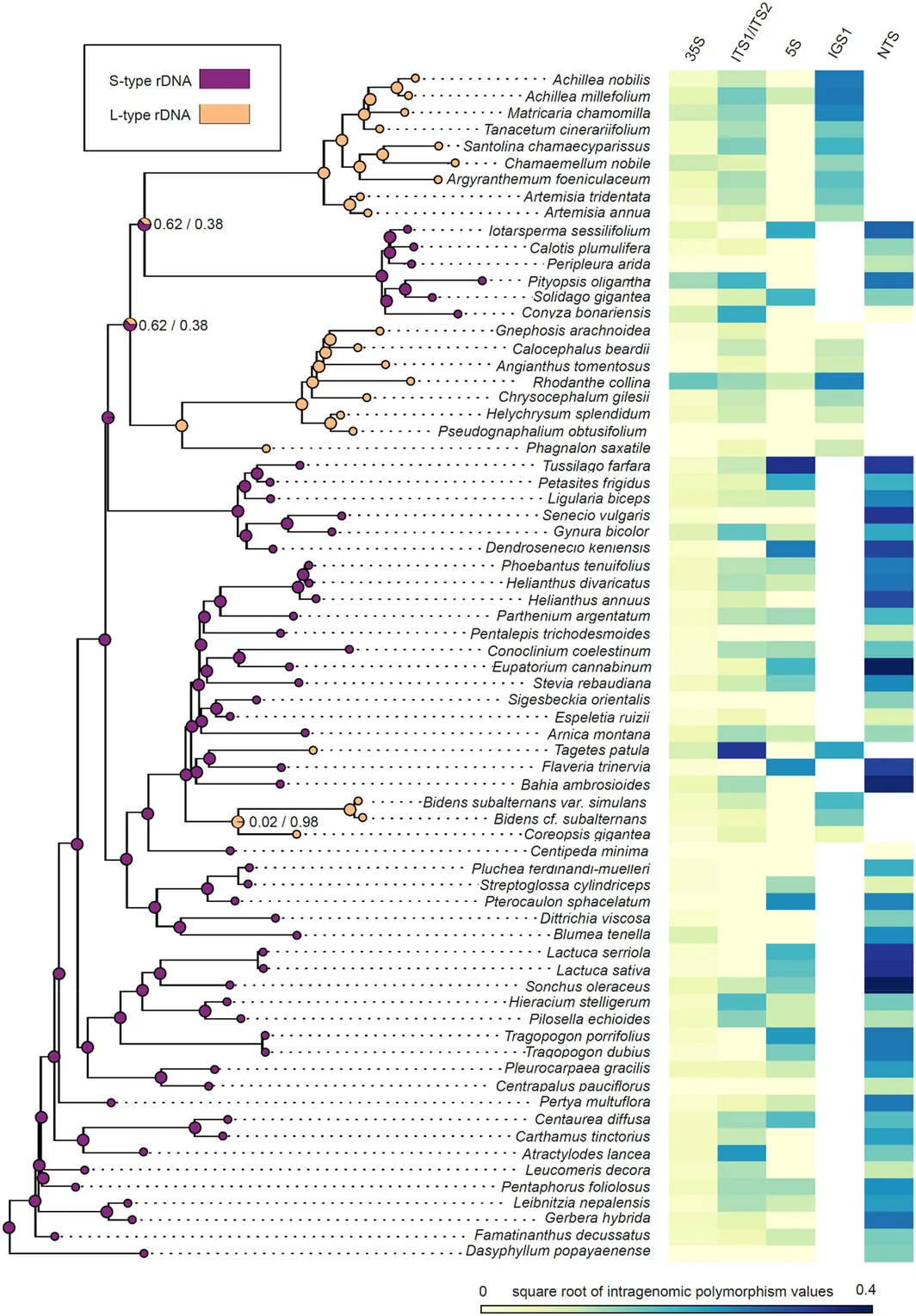
Ancestral character state reconstruction of 35S and 5S rDNA organization (L‐type or S‐type) on the 50% majority consensus tree from the Bayesian inference analysis. Node labels display the results of character reconstruction of rDNA organization (S‐type or L‐type) and the probabilities calculated across 100 replicates. Heatmap shows square root transformed values of intragenomic polymorphisms in 35S, ITS, 5S, IGS1, and NTS regions.

### Relationship between intragenomic variability of rDNA and its genomic proportion, copy number and number of rDNA sites

Spearman rank tests indicate a very strong correlation between the genome proportion estimated by a mapping approach and that from TAREAN clusters (Table S1), both for 35S and 5S rDNA (rho values >0.92; *P*‐values <0.0001). The genome proportion of 35S genic region including ITS (estimated from 5.8S mapping analyses, see section Methods) ranges between 0.047% (*Conoclinium coelestinum* DC., S‐type) and 3.44% (*Leucomeris decora* Kurz, S‐type) (Table S1), while the genome proportion of 5S genic region ranges between 0.0034% (*C. bonariensis*, S‐type) and 0.5620% (*T. farfara*, S‐type). The genomic proportion of 5S rDNA was significantly higher in S‐type than in L‐type species, with average values of 0.042% and 0.015%, respectively (χ^2^ = 8.324; *P* = 0.0039). In contrast, no significant difference was detected between L‐ and S‐type species for the genomic proportion of 35S rDNA, with average values of 0.662% and 0.566%, respectively. Regarding the relationship between intragenomic variability and the genomic proportion of rDNA (Table 3), Spearman rank tests do not show significant correlations between intragenomic polymorphism values and genome proportion of 35S (rho = 0.2237; *P* = 0.0607) or ITS (rho = 0.1930; *P* = 0.1069). However, the two species presenting the largest intragenomic variability (much larger than the rest) for 35S (*R. colina*) and for ITS (*T. patula*) are those showing the second and third largest 35S genomic proportions in our dataset. In contrast, for 5S, our results show a significant positive correlation between the intragenomic polymorphism values and the genome proportion of this region (rho = 0.4881; *P* <0.0001). The most extreme case is *T. farfara*, with an average 10x larger 5S genome proportion than the average of our dataset, being the species with the highest polymorphism values. Taking into account the phylogeny (Table 3), PGLS tests indicate a significant positive relationship between intragenomic polymorphism values and the genomic proportions of ITS (*P* = 0.0001) and 5S (*P* <0.0001). Pagel's λ analyses showed that the 35S genic region does not present a phylogenetic signal, so PGLS was not carried out for this region.

The copy number of 35S and 5S rDNA units was estimated for 44 species in our dataset for which published genome size values were available (Table S1). The copy number values for 35S rDNA show *c*. 8.6 fold variation, from 1469 copies (*Pentaphorus foliolosus* D.Don, S‐type, GS = 2.27 pg) to 12 704 copies (*Argyranthemum foeniculaceum* (Willd.) Webb ex Sch.Bip., L‐type, GS = 12.49 pg), with an average of 5154 copies. For 5S, the estimated copy number values show *c*. 167 fold variation, from 966 (*D. viscosa*, S‐type, GS = 2.30 pg) to 161 218 (*T. farfara*, S‐type, GS = 3.52 pg) with an average of 14 051 copies. The estimated copy number of 5S rDNA was also significantly higher in S‐type than in L‐type species (~18 200 versus ~6800 copies, respectively; χ^2^ = 5.60; *P* = 0.0179). In contrast, no significant difference was detected in 35S rDNA copy number between S‐type and L‐type species (~4700 versus ~6000 copies, respectively). Spearman rank tests do not detect a significant correlation between copy number and intragenomic polymorphism values for 35S or ITS regions (Table 3). In contrast, 5S shows a positive and significant correlation between copy number and intragenomic polymorphisms (rho = 0.4467; *P* = 0.0024) (Figure S5). Considering the phylogenetic relationships among the species, PGLS tests detect a significant and positive association between copy number and intragenomic ITS (*P* < 0.0001) and 5S (*P* < 0.0001) polymorphisms.

The number of rDNA sites, estimated from published rDNA‐FISH data, was available for 17 species from our dataset (Table S1). The reduced dataset prevented the use of PGLS analyses, so only Spearman rank correlations were conducted in this case (Table 3). Our results show that the number of 35S sites is significantly and positively correlated with the number of intragenomic polymorphisms of both 35S genic regions (rho = 0.5451; *P* = 0.0236) and ITS regions (rho = 0.6448; *P* = 0.0052). In contrast, the number of 5S sites detected by FISH shows a significant negative correlation with the intragenomic polymorphism values of the 5S genic region (rho = −0.5468; *P* = 0.0231).

## DISCUSSION

This work provides an extensive comparative analysis of rDNA arrangements and genomic homogeneity across Asteraceae, a family distinguished by exceptional diversity in rDNA organization. The occurrence of both linked and separated arrangements, sometimes even among close relatives, makes this group a particularly informative plant system for understanding how genomic context shapes patterns of rDNA evolution, while also offering insight into the role of concerted evolution and the dual gene–repeat nature of these loci.

### The evolutionary plasticity of rDNA organization is high: rDNA dynamics in family Asteraceae revisited

Although linked 35S–5S arrangements likely represent the ancestral state in plants, especially in bryophytes (Sone et al., 1999), most species have separate arrays, and this has been typically considered the rule. The discovery of the linked arrangement of rRNA genes in *Artemisia* (Garcia et al., 2009), later extended to several Asteraceae (Garcia et al., 2010), challenged that view. Those findings were accomplished using classical cloning, sequencing, Southern blot, and fluorescence in situ hybridization assays. Here, we revisit the high dynamism of rDNA organization using high‐throughput sequencing data and specific repeatome analyses. We refine the phylogenetic distribution of the linked arrangement reported in Garcia et al. (2010), where a single origin was hypothesized. Our study, using ancestral character state reconstruction of 35S and 5S rDNA organization (L‐type versus S‐type), infers up to four transitions from the S‐type to the L‐type (Figure 4). As proposed earlier and more recently by Maiwald et al. (2024), this pattern is consistent with the hypothesis of a single emergence of the 35S‐5S linked arrangement, followed by multiple losses and retentions, perhaps shaped by the hybridization and polyploidization evolutionary history of the Asteraceae tribes involved in L‐type and S‐type transitions (Huang et al., 2016; Watson et al., 2020). The similar 5S insertion and orientation patterns observed in the linked arrangement of those tribes, in contrast to the variability reported in other plants such as certain groups of gymnosperms (Garcia & Kovařík et al., 2013), showing L‐type rDNA, in which the 5S insertion appears either well within the IGS or very close to the 26S, and with 5S units in multiple orientations depending on the group, provide additional evidence to support the single origin of linkage in Asteraceae. To further test this hypothesis, we performed a phylogenetic reconstruction of 35S rDNA evolution (Figure S2) that places the tribes involved in L‐type and S‐type transitions within Asteroideae in an unresolved polytomy, contrasting with the good overall resolution elsewhere. Although the polytomy itself does not provide evidence for distinguishing between a single or multiple‐origin hypotheses, it is compatible with rapid diversification and hybridization, processes that Maiwald et al. (2024) invoke to explain the single‐emergence scenario.

Regardless of the origin, it is surprising that we still have not found compelling evidence of any species showing intermediate forms, where S‐type and L‐type coexist in the same genome. Earlier hints came from solo 5S‐hybridized bands in Southern blots or single 5S FISH signals (Garcia et al., 2010), and from 5S signals without 35S co‐hybridisation alongside linked loci (Abd El‐Twab & Kondo, 2007, 2009). Our data indicate complete homogeneity regarding one organization or the other, with two possible exceptions: *M. chamomilla* and *T. dubius*, in which an independent, small cluster with only 5S reads has been detected in both cases. BLAST suggests that the additional 5S cluster of *M. chamomilla* would correspond to a TRIM LTR retrotransposon, Cassandra, whereas the small 5S cluster in *T. dubius* likely represents a pseudogenized 5S array. Cassandra elements carry 5S sequences in their LTRs, can be tandemly arranged, and may coevolve with genomic 5S (Maiwald et al., 2024), but are probably unrelated to the emergence of the L‐type arrangement (Garcia et al., 2024; Maiwald et al., 2024). Occasional 5S solo signals in prior FISH (e.g. *M. chamomilla*, Table S1) or Southern blot analysis (see Figure S3) may therefore reflect Cassandra or a cluster of pseudogenized 5S rather than isolated 5S rDNA loci; however, this would need case‐by‐case confirmation.

The occurrence of linked and separated rDNA arrangements likely involves evolutionary trade‐offs. Linkage may help maintain stoichiometric balance of rRNA genes and promote uniformity in 5S rRNA (Garcia et al., 2012; Symonová et al., 2017), potentially improving translational accuracy and reducing ribosome heterogeneity. It may also face challenges like possible transcriptional interference between Pol I and Pol III, constrained evolutionary flexibility of 5S genes, and perhaps greater genomic fragility if NORs are damaged or silenced. Separate arrangements provide regulatory independence and may enable the evolution of specialized 5S rRNA variants, which could be evolutionarily advantageous (e.g., in complex life cycles or tissue‐specific contexts; Davidian et al., 2022). On the negative side, they may risk imbalanced rRNA production, increased regulatory complexity, and ribosome heterogeneity if not tightly controlled. Nevertheless, both configurations persist in nature, possibly shaped by their particular adaptive value in specific genomic and/or developmental contexts.

### Intragenomic rDNA diversity in Asteraceae: Revisiting the model of concerted evolution

Concerted evolution is the genomic mechanism by which repetitive DNAs, such as ribosomal RNA genes, are homogenized within a genome so that all copies remain very similar or even identical. In theory, concerted evolution should eliminate sequence differences among the hundreds or thousands of rDNA repeats in an individual. We analyzed rDNA polymorphisms in Asteraceae across all regions in S‐ and L‐type species. The results reveal striking contrasts in sequence diversity between both groups, provide insight into rDNA dynamics, and call for a more nuanced interpretation of concerted evolution, particularly regarding 5S rRNA genes.

The coding sequences of rDNA within a genome are typically highly homogeneous, due to concerted evolution with purifying selection removing deleterious variants (Sultanov & Hochwagen, 2022). Once a mutation arises, repeated rounds of unequal crossover or gene conversion generate chromosomes with increased or decreased numbers of variants. Chromosomes containing certain frequencies of mutations within the rRNA genes are usually purged (or kept at low frequency) because of strong functional constraints, while mutations in the noncoding spacers face weaker constraints (Eickbush & Eickbush, 2007). Thus, intragenomic polymorphism in rDNA genic regions is generally much lower in genic than in spacer regions.

Our dataset confirms this statement to a large extent for 35S rDNA. Intragenomic variability in the ITS is ca. 6‐fold higher than in the 18S, 5.8S, and 26S genic regions (Table 1), comparable to other taxa surveyed with similar high‐throughput sequencing approaches. For instance, *Arabidopsis thaliana* (L.) Heynh., *Nicotiana tomentosiformis* Goodsp., the moss *Physcomitrella patens* (Hedw.) Bruch & Schimp., and the liverwort *Marchantia polymorpha* L. showed 2‐ to 6‐fold more variability in ITS than 35S genic regions (Rosato et al., 2016). In *Drosophila* (Stage & Eickbush, 2007), the coding and noncoding regions had similar levels of variants present in the low‐frequency class, but for the high‐frequency variants, the noncoding regions had 12 times the number of variants present in the coding region, consistent with selection limiting expansion of coding variants. In *Asclepias* (Weitemier et al., 2015), highly polymorphic sites are dramatically less frequent in genic than spacer regions, proposing that this selection pressure could be positively correlated with the proportion of rDNA copies. In Asteraceae, differences between coding and noncoding regions are also much greater for high‐frequency polymorphisms, underscoring the role of purifying selection (and copy number; see below) in shaping the homogenization efficiency of concerted evolution. Different technical approaches can shift absolute estimates, so cross‐study comparisons require caution. Nonetheless, many eukaryotes harbor measurable intragenomic diversity in 35S repeats, and complete identity is rarely achieved.

L‐type species show higher—although not significantly—polymorphism rates than S‐type species both in 35S genic/coding (2.28‐fold) and ITS/non‐coding (1.67‐fold) regions. Conversely, the 5S genic region displays a very low level of polymorphisms in L‐type species (average 0.08), as compared to S‐type species (average 1.87), which represents more than a 23‐fold higher level of polymorphisms (Table 2; Figure 3). Most L‐type species have no detectable 5S variants among hundreds to thousands of copies. Only two L‐type taxa, *A. millefolium* and *R. collina*, had a single nucleotide polymorphism in the 5S coding region. This near‐identity spans at least four different tribes of Asteraceae diverging ca. 35–20 Mya (Mandel et al., 2019), implying parallel outcomes of the L‐type arrangement.

A possible explanation for the uniformity of 5S in L‐type species could be related to the likely evolutionary recent integration(s) of the 5S rRNA gene into the 35S spacer in Asteraceae (Garcia et al., 2010). If a single event linked 5S to 35S, this could be comparable to a “founder effect”, and in this scenario, only a particular 35S‐5S monomer would become the “template” for all repeats thereafter. With the strong homogenizing pressure of concerted evolution, that specific sequence would be replicated with near‐perfect identity. And given that the integration of 5S into 35S may have happened recently in evolutionary terms, there would have been limited time for new mutations to accumulate. However, we found a similar intragenomic variability of 35S (including genic and spacer regions) in L‐type species and S‐type species (Table 2; Figure 3), so it is hard to attribute the lower number of polymorphisms in 5S genes to the recent origin and replication of the linked 35S‐5S “template”; i.e., unequal crossing over or gene conversion should similarly affect 35S and 5S linked genes.

A more plausible hypothesis is that linkage to the 35S array promotes more effective homogenization of 5S repeats through concerted evolution, potentially facilitated by biased recombination events and reinforced by purifying selection. Because 35S and 5S are physically linked in L‐type species, unequal crossing over and gene conversion are expected to homogenize both arrays, including coding and non‐coding regions, while selection may differ among regions. Indeed, 35S genes, ITS, IGS, and 5S genes of L‐type species show contrasting polymorphism levels despite being linked in the same unit (Table 2). Therefore, our results suggest that 5S genes would be subject to stronger selection pressure than 35S genes when organized in an L‐type configuration. By contrast, 5S arrays in S‐type species accumulate much higher intragenomic diversity (Figure S6), exceeding most intergenic/non‐coding regions analyzed and surpassed only by their spacer, the NTS (3.2‐fold higher). Overall, considering genic and non‐genic sequences, 5S in S‐type species is much more prone to polymorphisms than 35S.

Previous studies reported lower sequence homogeneity of separate 5S versus 35S arrays in many organisms (Garcia et al., 2017; Symonová et al., 2017; Vierna et al., 2013; Vizoso et al., 2011) and invoked birth‐and‐death evolution (Hughes & Nei, 1989; Nei & Rooney, 2005; Vierna et al., 2009). By this model, genes evolve through repeated cycles of duplication (birth) and pseudogenization or deletion (death). Different from concerted evolution, which tends to sequence homogenization, birth‐and‐death allows for retention of diverged paralogs, leading to sequence diversity among gene copies both within and between genomes. Several studies have explained 5S's lack of sequence homogeneity under these terms (Pinhal et al., 2011; Volkov et al., 2017), although this can also be interpreted as lower or less efficient concerted evolution (Wang et al., 2023). But why does this happen much more to 5S than to 35S? Some hypotheses may explain it: (1) The degree of repetitiveness is higher (about threefold) for 5S than 35S rDNA. Probably due to its reduced size, there are many more copies of 5S (around three times more in our whole dataset) than of 35S genes, and larger arrays (in terms of monomer numbers) increase the chance of accumulating pseudogenes or divergent copies – mostly in low frequency‐ by skipping the homogenizing action of purifying selection (but see below). The higher abundance of structural variants (more possibly representing non‐functional variants than those with single nucleotide polymorphisms) in 5S than in 35S would also support the hypothesis that selection is more relaxed on 5S in S‐type species; (2) Different from 35S loci, which are typically located terminally in chromosomes, 5S loci tend to locate interstitially or pericentromerically (Garcia et al., 2017). Lower meiotic recombination (hence less homogenization) in interstitial than in terminal regions is a widespread pattern in many taxa. This could also partly explain the higher divergence of 5S arrays in S‐type than in L‐type species, where 35S‐5S loci tend to be located terminally.

Spacer regions with measurable polymorphisms (NTS for 5S rDNA and IGSi for 35S rDNA in S‐type species; IGS1 and IGSi2 for L‐type species; see Figure 1 and Table 2 for details) show much higher variation than genic regions, as expected (except for the 5S gene in S‐type species as discussed above), reflecting weaker functional constraints (e.g. Sultanov & Hochwagen, 2022). This is particularly true for the NTS of the 5S gene in S‐type species, which presents the highest variation rate of all regions here analyzed (average 5.99 polymorphisms/100 bp). If purifying selection—as the last necessary step of concerted evolution—affecting 5S of S‐type species is moderate to low, the NTS might escape homogenization more readily. In L‐type species, the equivalent region IGS1 (see Figure 1) also presents a relatively high level of variation (an average of 3 polymorphisms/100 bp), being the second most variable region of our dataset after the NTS. Besides, IGSi (in S‐type) and IGSi2 (in L‐type) have been taken as a proxy to the variability of the large IGS connecting the rDNA genic blocks one after the other. As explained earlier, addressing the polymorphism rate in the IGS is challenging due to the highly repetitive nature of this region, so only the initial part has been considered. Interestingly, their average polymorphism rate is comparable (1.51 versus 1.83 in S‐type versus L‐type species), suggesting that selection pressure could be acting with similar intensity in these two functionally equivalent regions, also showing comparable copy numbers.

### Copy number, loci number and genome organization as key factors of concerted evolution

The dynamics and outcome of concerted evolution of rRNA genes depend not only on unequal crossing over, gene conversion and selection but also on factors related to genomic architecture, such as copy and locus number. Theoretical models predict that the time required for homogenization increases roughly linearly with array size (Smith, 1976), making copy number a critical factor. Besides, homogenization is slower (or polymorphisms are much more frequent) across multiple loci than within a single large array (e.g., Dover, 1982; Eickbush & Eickbush, 2007). For instance, 5S rDNA families located on different chromosomes described in *Rosa* (Vozárová et al., 2021) have evolved independently over millions of years.

Beyond sequence variation, Asteraceae also differ in rDNA copy number by genomic organization. As expected, L‐type species tend to have a more balanced number of 35S and 5S repeats: on average, ~6000 copies of 35S and ~6750 copies of 5S. Conversely, S‐type species often have disproportionately many more 5S than 35S copies, roughly four times more on average (~18 200 copies of 5S versus ~4600 copies of 35S). These differences suggest that when 5S is in a separated configuration, it can amplify in copy numbers without the same constraints as when it is linked to 35S. But, as discussed earlier, a higher copy number might come at the cost of accumulating more variants, potentially including non‐functional ones. In fact, across Asteraceae, we found a significant positive correlation between 5S rDNA copy number and the level of intragenomic polymorphism in the 5S gene (Table 3; Figure S5). In contrast, no significant correlation was observed between 35S rDNA copy number and the level of 35S intragenomic variability. This, again, points to the role of selection in maintaining the homogeneity of rDNA genic regions. The copy number of the 35S genes would be better adjusted to the biological needs of the plant, and therefore selection would act quickly, eliminating individuals with a relatively high number of mutated, non‐functional copies. On the other hand, 5S rDNAs of S‐type species, with several times more copies than the 35S, would accumulate variants (pseudogenised genes) without fitness being affected (because there are also many functional ones available) and without selection acting to eliminate those individuals carrying mutated copies.

Another factor potentially influencing rDNA homogeneity is the number of chromosomal rDNA sites in a given genome (Wang et al., 2023). Species with a high number of rDNA loci are more likely to display high intragenomic variability levels since interlocus homogenization is less frequent than intralocus homogenization (e.g., in *Asclepias*, Weitemier et al., 2015; Arabidopsis, Riddle & Richards, 2005; *Nicotiana*, Matyášek et al., 2012; and *Ephedra*, Wang et al., 2016). In Asteraceae, 35S intragenomic variability increases with the number of sites. By contrast, 5S variability shows a negative correlation with site number, driven mostly by L‐type species, which average 8.6 5S signals versus 2.6 in S‐type species, yet exhibit almost no 5S variability. Locus‐number data are sparse, so this pattern needs confirmation.

The intriguing question is why most genomes carry many more 5S than 35S copies. Multiple factors may contribute: (i) the genomic architecture of 5S rDNA, with often smaller arrays spread across multiple sites, can favor copy‐number gain via extrachromosomal circles that reintegrate at new sites (Cohen et al., 2010); (ii) the shorter repeat unit of 5S means that many more repeat units are included in a 5S array than in a 45S array in a given physical length. More repeat units create more potential alignment mismatches during meiosis or DNA repair, increasing the chances of unequal crossover events that most likely add (but may occasionally delete) repeats, especially since expansions are often neutral while deletions may be selected against (Ganley & Kobayashi, 2011; Jeffreys et al., 1998; Kobayashi & Sasaki, 2017; Smith, 1976); (iii) a compensatory amplification of 5S rDNA driven by Polymerase III capacity: 45S rDNA is transcribed by RNA Polymerase I, and up to hundreds of molecules of this enzyme can simultaneously transcribe a single rDNA unit (Azouzi et al., 2026), whose transcribed region is 8.4 kb in Arabidopsis (Sáez‐Vásquez & Delseny, 2019) and whose IGS is highly variable, from around 1 to ca. 23 kb long in plants (Coutinho et al., 2016; Rogers & Bendich, 1987). Conversely, 5S rDNA is transcribed by RNA Polymerase III, and given its 120‐bp long size, only a single 5S rDNA copy can be processed at a time (Dieci & Sentenac, 2003). Polymerase III is also physically bigger than Polymerase I (Khatter et al., 2017). Therefore, it is plausible that more 5S rDNA “templates” would be needed to match the different transcriptional capacities of Pol I and Pol III. In other words, having a higher copy number of 5S rDNA could ensure that 5S rRNA does not become limiting for ribosome assembly.

## CONCLUSION

Our results show that Asteraceae provides an informative model for understanding ribosomal DNA evolution more broadly. Within a single angiosperm family, it captures repeated shifts in rDNA organization, contrasting degrees of sequence homogenization and a strong effect of genomic context on the evolutionary fate of ribosomal repeats. More generally, our findings indicate that concerted evolution is not a uniform process leading inevitably to sequence identity, but a context‐dependent outcome shaped by the interaction between homogenizing mechanisms, purifying selection, and genome architecture.

Eickbush and Eickbush (2007) portrayed concerted evolution of rRNA genes as a finely tuned orchestra led by unequal crossing over and gene conversion, with selection shaping the outcome. Our results stress the importance of selection and show that the orchestra's stage also matters: genome architecture (copy number, number of loci and chromosomal position) determines whether 5S plays in unison or not. L‐type genomes keep 5S in a tight homogenized ensemble, while S‐type genomes allow multiple 5S soloists. The core message is that genome architecture largely determines intragenomic rDNA diversity, making the degree of concerted evolution context‐dependent rather than universal.

## MATERIALS AND METHODS

### Sampling

We used whole‐genome sequencing data from 71 species of Asteraceae, belonging to 20 tribes and representing 8 subfamilies, with a particularly well‐represented sampling from subfamily Asteroideae, where both linked (L‐type) and separate (S‐type) arrangements coexist. Nine species were specifically sequenced for this study while the remaining 62 were represented by existing genomic datasets (available through repositories such as NCBI and ENA). Data were carefully curated to select high‐quality genomic assemblies with comprehensive coverage of rDNA loci. A list with details on the species selected for this study can be found in Table S1.

### DNA extraction and sequencing

The total DNA of nine species (Table S4) was isolated from dried leaf material using either a modified CTAB protocol (Doyle & Doyle, 1987) or the E.Z.N.A® Plant DNA Kit (Omega Bio‐tek, Inc., Norcross, GA, USA), depending on the quality and/or sufficient amount of material. Herbarium vouchers of the specimens are deposited at the Institut Botànic de Barcelona (IBB, CSIC‐CMCNB). The quality of each extraction was checked by spectrophotometry with NanoDrop 1000 (PeqLab, Erlangen, Germany) and the DNA concentration by fluorometry with Qubit Fluorometric Quantification (Thermo Fisher Scientific, Waltham, MA, USA). De novo random sequencing of whole DNA was performed by Beijing Genomics Institute (BGI; Shenzhen, China), employing an Illumina HiSeq X10 (Illumina, San Diego, CA, USA) platform, generating around 10 million paired‐end reads (150 nt long) from ~500 bp insert‐size fragment libraries (Table S4). To obtain a reference of 35S and 5S genes, whole‐genome resequencing of *A. annua* was also performed using the PacBio RSII platform (20 kb library preparation and sequencing on two SMRT Cells, generating approximately 2 Gb of data with average read lengths of 8–10 kb) at a depth of 1× coverage (SRR37646768‐69).

### Southern blot hybridization

Purified genomic DNAs of 11 species of the dataset were digested with BamHI restriction enzyme and resolved by electrophoresis in 0.9% (w/v) agarose gels. Following separation, DNA was transferred to Hybond‐XL nylon membranes (GE Healthcare, Little Chalfont, UK) by alkaline blotting and hybridized with ^32P‐labeled probes generated using the DekaLabel kit (MBI/Fermentas, Vilnius, Lithuania). Membranes were washed at high stringency, and hybridisation signals were detected with a Phosphor Imager (Typhoon 9410; GE Healthcare, PA, USA). Band intensities were quantified using ImageQuant software (GE Healthcare). Probes derived from a tomato 26S rDNA probe (X13557.1) from Prestle et al. (1992) and from an *A. tridentata* rDNA probe (JX101915.1) from Garcia et al. (2012).

### rDNA reconstruction and intragenomic variation analyses

The identification and reconstruction of rDNA genes was performed with TAREAN (Novák et al., 2017), a specific tool for the analysis of tandem repeats implemented in RepeatExplorer2 (Novák et al., 2013), running on the GALAXY server (https://galaxy‐elixir.cerit‐sc.cz). The fastq reads were initially filtered for quality and trimmed to a uniform length by pre‐processing and QC tools by RepeatExplorer2, following the recommendations of the authors (Novák et al., 2020). After converting filtered FASTQ reads to interlaced FASTA format, individual TAREAN analyses were carried out for each accession employing default clustering settings (90% sequence similarity spanning at least 55% of the read length), allowing the cluster merging (‐‐merge_threshold 0.1) and the automatic filtering of abundant satellite repeats (‐‐automatic_filtering) to allow more reads to be analyzed and increase the chances of reconstructing the rDNA. We used the maximum number of randomly sampled reads that could be processed by TAREAN implemented on the GALAXY server using the ‘large and long’ option (Table S1). Plastid and mitochondrial DNA reads were removed prior to downstream analyses.

The consensus sequences of rDNA, together with their genomic relative abundance, were extracted from the TAREAN output report for each analyzed species. The different regions constituting the rDNA units were annotated in Geneious Prime 2023.0.4 (Biomatters Ltd, Auckland, New Zealand) using as reference the 10 370 bp‐long 35S‐5S unit from *A. annua*. For annotation, each species‐specific rDNA consensus sequence was imported into Geneious and pairwise aligned to the *A. annua* 35S–IGS1–5S–IGS2 reference (see below). Feature boundaries (18S, ITS1, 5.8S, ITS2, 26S, ETS/IGS segments and the 5S gene) were initially projected from the reference based on sequence homology across the alignment (Geneious “transfer/propagate annotation” workflow) and subsequently curated manually. Manual curation focused on adjusting region limits to the nearest supported alignment breakpoints and conserved 5S gene sequences, ensuring that spacer regions (ITS and IGS) were delimited by the ends of the flanking coding regions rather than by arbitrary length cut‐offs. The reconstruction of the 35S‐5S rDNA *A. annua* reference was carried out by combining RepeatExplorer2 (RE) clustering of Illumina reads and long PacBio reads. Briefly, the contigs from RE clusters no. 19, 20 and 24 were assembled using the “Add sequences to reference” function in CLC Genomics Workbench (QIAGEN, Aahrus, Denmark), (CLC onwards) using the following alignment parameters: minimum aligned read length_100 bp, alignment stringency_medium, and the longest CL19 as the reference. The resulting 6416 bp‐long contig was then BLASTed against the PacBio reads from *A. annua* and the reads with highest scores were extracted. Ten overlapping PacBio reads were aligned with the RE 6416 bp contig. The full length rDNA units were identified using the dotplot self‐to‐self comparisons of analysis of individual PacBio reads. The repeats were regularly spaced at about 10 kbp. Five reads with longest overlaps were aligned and a consensus sequence generated. The correct order of genes were checked by blasting of the consensus sequence against the 18S and 26 rDNA reference sequences (GenBank X51576.1, X13557.1 accessions). The 10 370 bp full length 35S‐IGS1‐5S‐IGS2 consensus monomer was then polished by short Illumina reads.

Polymorphism quantification was performed by mapping analysis using CLC. Trimmed reads of each species (Table S1) were mapped to the corresponding annotated consensus sequence with following parameters: mismatch cost = 2, insertion and deletion costs = 3, length fraction = 0.8, similarity fraction = 0.8. Variations were then detected via the Low Frequency Variant Detection tool in CLC, using default settings. Single nucleotide variants (SNVs), also called single nucleotide polymorphisms (SNPs), were filtered as follows: coverage ≥100, count (the number of countable reads supporting the allele) ≥20, frequency (the ratio of the number of ‘countable’ reads supporting the allele to the number of ‘countable’ reads covering the position of the variant) ≥5%. Other variation types, i.e. deletion, insertion, replacements (i.e. one or more bases replaced by one or more bases, where the identified allele has a length different from the reference) and multiple nucleotide variants (i.e., two or more SNVs in succession; MNV), were produced by default settings.

The mapping analysis of the 35S intergenic spacer of S‐type species and the 5S‐35S intergenic spacer of L‐type species (termed IGS and IGS2, respectively Figure 1) revealed the presence of multiple subrepeats, preventing the correct estimation of the variants along the complete range of these regions. Therefore, only the initial fragment at the 3′ end of 26S (in IGS) or the 5′ end of 5S (in IGS2), showing no evidence of subrepeats presence, was considered for downstream analyses. In the case of L‐type species, a fragment of 122 bp (i.e. selected for comparable reasons as the minimum length of NTS in S‐type species) next to 5S gene was annotated as a representative portion of IGS2 (termed IGS2i; see Figure 1) and included in further analyses. In the case of S‐type species, a fragment of 131 bp (i.e. the minimum length of IGS1 observed in our Asteraceae dataset) at the 3′ end of 26S gene was annotated as representative of IGS (termed IGSi; see Figure 1) and retained for subsequent analyses. The frequency of intragenomic variants for each rDNA region was calculated as variants per 100 nucleotides, considering all types of nucleotide and structural polymorphisms unless otherwise stated, and accounting for the length of the regions in the annotated consensus rDNA sequences of each species.

### Plastome reconstruction

Plastid genome reconstruction was performed with a mixed strategy combining de novo reconstruction of all plastomes and mapping assemblies of reads. De novo reconstruction of plastid genomes was performed through the NOVOPlasty pipeline version 4.3.1 (Dierckxsens et al., 2017), using the raw whole dataset of Illumina reads ‐the same datasets employed for rDNA analyses‐ previously trimming the adapters, as recommended by the authors. Plastid genome assembly for each accession was done using as seed the rbcL gene of the same species retrieved from NCBI GenBank and, if the assembly was incomplete, the process was repeated using a different seed sequence (e.g. the matK gene, or the entire chloroplast sequence obtained from NCBI Genbank). The resulting contig options were aligned to the previously published plastome genome of *Artemisia giraldii* Pamp. (NCBI GenBank: OK128342) using MAFFT (Katoh & Standley, 2013) and manually rearranged so that the short single copy and inverted repeat were in the same orientation for each individual using Geneious Prime v. 2023.2.1. Then, filtered whole genome sequencing reads for each species with a minimum quality score of 30 were mapped to the consensus plastome sequence using bwa (Li & Durbin, 2009), and the resulting consensus, with a minimum coverage of 20 reads per site, was manually adjusted for further phylogenetic analyses. All sites not supported by 90% of the mapped reads were replaced by Ns.

The plastomes of *Espeletia cardonae* Cuatrec. (tribe Millerieae), *Pentalepis trichodesmoides* F.Muell. (tribe Heliantheae), and *Dasyphyllum popayanen*se (Hieron.) Cabrera (tribe Barnadesieae) were not successfully reconstructed using the corresponding datasets of Illumina reads employed for rDNA analyses. For *E. cardonae*, the draft plastome sequence of the closely related species *E. ruizii* Cuatrec. from Pouchon et al. (2018) was downloaded and employed as a reference to map filtered Illumina reads of the same species (EBI ERR4604241), subsequently building a consensus plastome sequence following the above‐mentioned approach. For *P. trichodesmoides*, we were not able to find any published plastid genome for this taxon or for species from the same genus, so the plastome of *Dimerostemma asperatum* S.F.Blake—a closely related species within the same subtribe, Ecliptinae—was downloaded from NCBI Genbank (MT700540) and employed for further analyses. For *D. popayanense*, the published plastome of the same species available in NCBI Genbank (OR069729) was downloaded and employed for subsequent analyses.

Each resulting plastome was annotated using the software GeSeq (Tillich et al., 2017) included in the platform MPI‐MP CHLOROBOX (https://chlorobox.mpimp‐golm.mpg.de), selecting the options to perform ARAGORN v. 1.2.38 and BLAT, as recommended by authors for plastomes. Subsequently, all automatically annotated consensuses were checked manually using Geneious.

### Phylogenetic analysis

Plastid phylogenetic tree was inferred after removing one of the inverted repeats and the fragment of the plastomes containing a double inversion located in the long single‐copy region of all Asteraceae subfamilies excepting Barnadesioideae (Kim et al., 2005). The genic regions of the plastomes were extracted and subsequently partitioned on tRNA, rRNA and CDS regions. The resulting sequences were aligned using MAFFT (Katoh & Standley, 2013) and PartitionFinder2 (Lanfear et al., 2017) was employed to fit the best nucleotide substitution model for all the different partitions. Bayesian inference phylogenomic tree was constructed using MrBayes v. 3.2.6 (Ronquist et al., 2012), with two independent Markov chains Monte Carlo (MCMC) run for 1 000 000 generations, and tree sampling every 1000 generations. The average standard deviation was confirmed to be less than 0.01 at the end of the analysis, and the potential scale reduction factor was near 1.0 in all parameters. The first 25% of the trees were discarded as “burn‐in,” and the posterior probability was estimated by constructing 50% majority‐rule consensus tree. Maximum likelihood (ML) phylogenomic tree was also constructed from the same dataset, using the program RAxML version 8.2.10 (Stamatakis, 2014) with 1000 bootstrap replicates, the GTR + G + I substitution model for all partitions and other parameters using the default settings. Branches with bootstrap support <50% were collapsed into polytomies. Phylogenomic trees were visualized with FigTree v. 1.4.5 (https://tree.bio.ed.ac.uk/software/figtree/), using as an outgroup *Gamocarpha macrocephala* S.Denham & Pozner (Calyceraceae).

The phylogenetic reconstruction of 35S rDNA evolution was inferred from the consensus sequences of TAREAN after removing the IGS regions as well as the 5S gene in the case of L‐type species. The sequences were partitioned among rRNA and ITS regions, subsequently aligned using MAFFT. PartitionFinder2 was employed to fit the best nucleotide substitution model for all the different partitions. Bayesian and ML phylogenetic analyses were performed following the same approaches as for the plastid evolutionary reconstructions.

### Statistical analysis and phylogenetic comparative analysis

Data manipulations and statistical analyses were performed with RStudio 2024.04.2 (http://www.rstudio.com/), a user interface for R 4.4.1 (R Core Team, 2024). Given that our datasets were not normally distributed, we used nonparametric tests such as Spearman rank correlation and the Kruskal–Wallis test by ranks to investigate the relationship among rDNA intragenomic variability and other genomic variables as rDNA arrangement (S‐type or L‐type), rDNA genomic proportion, rDNA copy number, or number of rDNA signals detected by FISH. The organization of rDNA regions (S‐type or L‐type) in the species of our dataset was determined according to the reconstructions obtained in TAREAN. The genome proportion of 5S rDNA was estimated from the ratio of the number of reads mapped to the consensus sequence of 5S gene and divided by the total number of reads analyzed. In the case of 35S, the genome proportion was estimated from the ratio of the number of reads mapped to the consensus of 5.8S gene (taking the latter as a proxy of 35S rRNA), divided by the total number of reads and scaled to the length of 35S genic region. The rDNA copy number of 5S and 35S was calculated for those species in our dataset with available genome size information, using the formula employed in Wang et al. (2016): i.e. copy number = genome proportion × genome size/gene length. The nuclear DNA content of 12 species was assessed using flow cytometry in a CyFlow® Space flow cytometer (Sysmex, Chuo‐Ward, Kōbe, Japan) at Institut Botànic de Barcelona (Table S5). The methodology detailed in Garcia et al. (2004) was followed, using *Pisum sativum* L. cv ‘Express Long’ (8185.86 Mb/2C) as the internal standard. Genome size values of the other species were obtained from the GSAD database 3.0 (Garnatje et al., 2011), unless other sources are indicated in Table S1. The number of FISH signals of the species in the dataset was obtained from the Plant rDNA database 4.0 (Rodríguez‐González et al., 2023) unless other sources are indicated in Table S1.

To analyze relationships among rDNA intragenomic variability, rDNA genomic architecture (L‐type versus S‐type), rDNA genomic proportion and rDNA copy number in a phylogenetic context, the phylogenetic generalized least squares (PGLS) algorithm, as implemented in the ‘nlme’ package for R, was employed.

The phylogenetic signal of 35S, 5S, ITS (including ITS1 and ITS2) and NTS intragenomic variability was estimated using an evolutionary approach [i.e. Pagel's λ (Pagel, 1999)] with ‘phylosig’ function on phytools 2.0 package (Revell, 2024) in R. We also reconstructed the evolution of S‐type and L‐type rDNA organization in our Asteraceae dataset with a stochastic character mapping (SIMMAP) analysis (Bollback, 2006), using the ‘make.simmap’ function in phytools package and 100 simulations. An Equal Rates model (ER) was selected after comparison with other models (ADR and SYM) by calculating their AIC values with ‘fitMk’ function. The estimated node and tip states were mapped onto the phylogenetic tree resulting from the MrBayes analysis with the ‘plotTree’ function. Intragenomic variability (including nucleotide and structural polymorphisms) of 35S, ITS1‐ITS2, 5S and NTS/IGS1 regions among species was illustrated by constructing a phylogenetic heatmap, with square root transformed polymorphism values for better visualization, as implemented in ‘phylo.heatmap’ function of phytools package.

## CONFLICT OF INTEREST

The authors declare no conflicts of interest.

## Supporting information


**Figure S1.** Bayesian phylogenetic tree of Asteraceae based on plastome sequences.
**Figure S2**. Maximum‐likelihood phylogenetic tree of Asteraceae based on plastome sequences.
**Figure S3**. Bayesian phylogenetic tree of Asteraceae based on 35S rDNA sequences.
**Figure S4**. Maximum‐likelihood phylogenetic tree of Asteraceae based on 35S rDNA sequences.
**Figure S5**. Southern blot hybridisation of BamHI‐digested genomic DNA from selected Asteraceae species showing contrasting rDNA arrangements.
**Figure S6**. Scatterplots showing the relationship among intragenomic polymorphism values, copy number and genome proportion in 5S, 35S and ITS rDNA regions.
**Figure S7**. Boxplots of intragenomic polymorphism values in 35S and 5S rDNA regions among species showing different rDNA arrangements.
**Table S4**. Collection details and herbarium information of the species sequenced on this study.
**Table S5**. Genome size estimations of Asteraceae species sequenced.

**Table S1.** Details on the species selected for this study, basic genomic data and summary of rDNA variability.

**Table S2.** Length of the genic and intergenic rDNA regions reconstructed for each Asteraceae species.

**Table S3.** Raw variability values calculated for each rDNA region and for each Asteraceae species included in the study.

**Data S1.** Concatenated alignment of Asteraceae plastome sequences employed in phylogenetic analyses.

## Data Availability

New sequencing data is available at the National Center for Biotechnology Information (NCBI) library under the accession PRJNA764566 (https://www.ncbi.nlm.nih.gov/bioproject/PRJNA764566/) titled “Deconstructing ribosomal DNA: from the sequence to the chromosome across the Tree‐of‐Life (rDNA‐LIFE)”.
