# Supplementary material for: Genome architecture modulates 35S and 5S rDNA homogenization in Asteraceae
Source: Plant J. 2026 Oct 22;128(2):e71174. doi: 10.1111/tpj.71174 (PMC13688046; doi:10.1111/tpj.71174)
Supplement: Supplementary file 1 [file TPJ-128-0-s004.pdf]

Supporting Information – Vitales et al. 2026. Genome architecture modulates 35S and 5S rDNA homogenization in Asteraceae

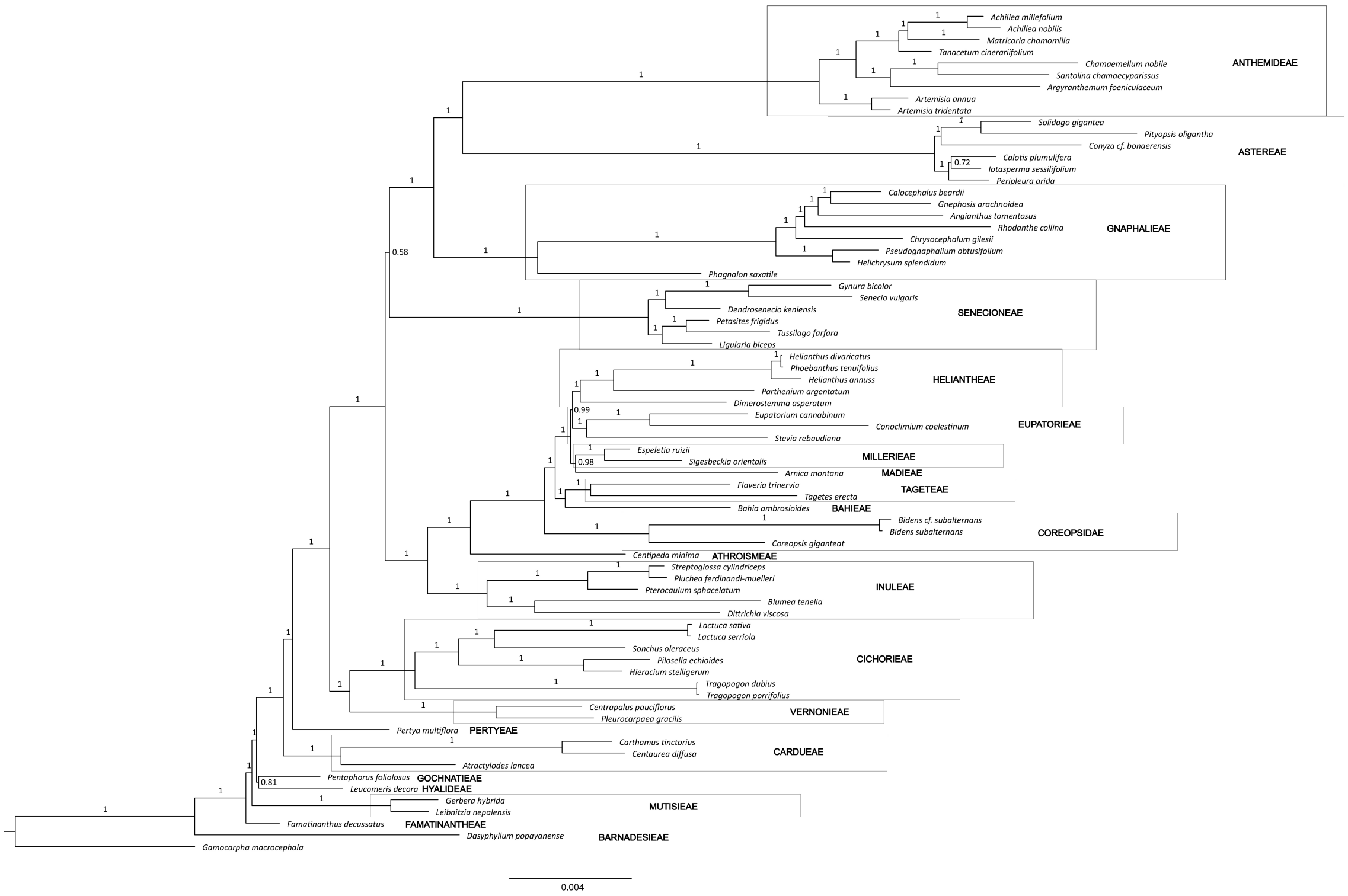

Figure S1. Phylogenetic tree of Asteraceae obtained using Bayesian inference, based on plastome sequences of tRNA, rRNA and CDS regions. Tribes are indicated beside the tree. The numbers above the branches indicate posterior probability.

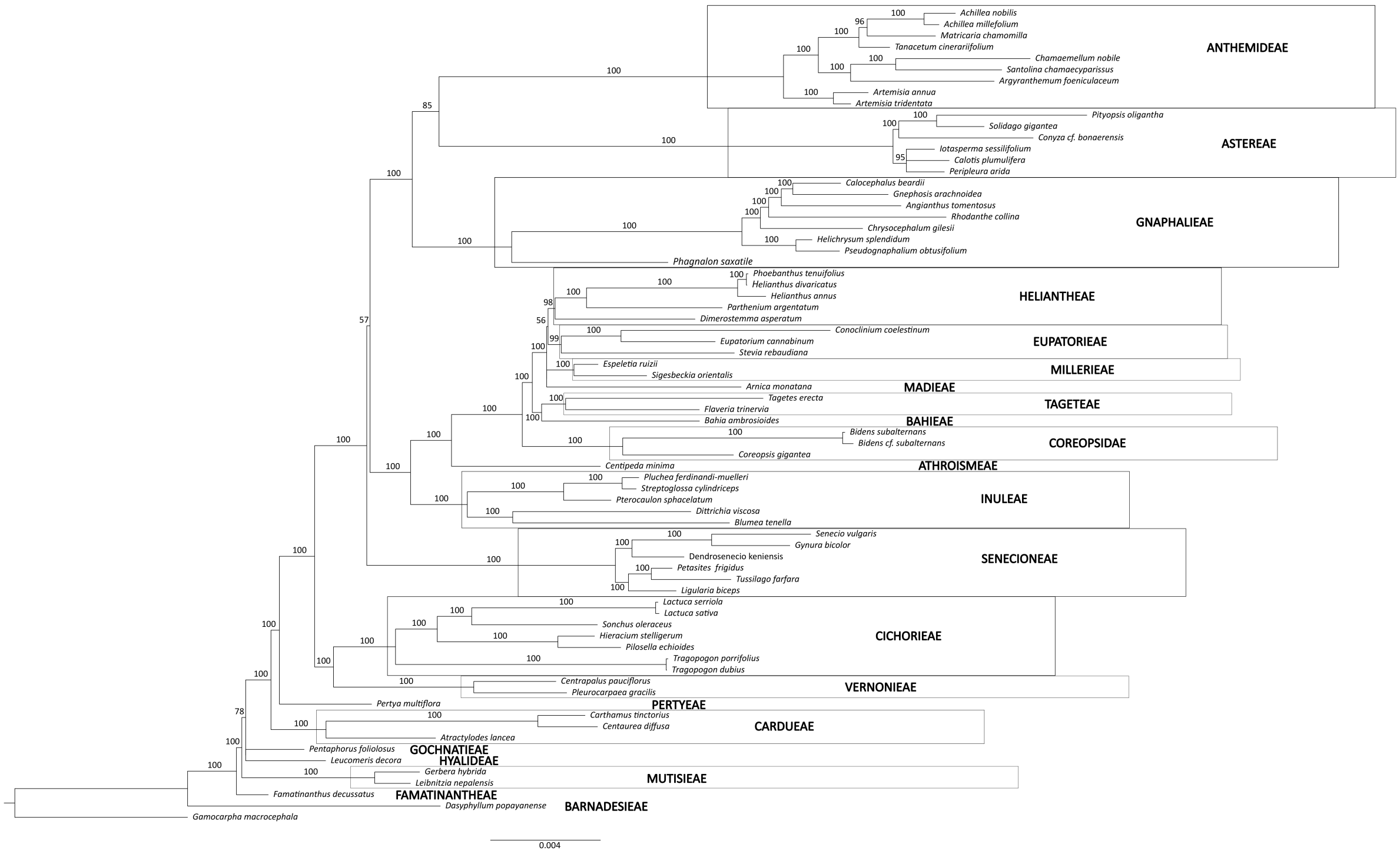

Figure S2. Phylogenetic tree of Asteraceae obtained using Maximum-likelihood inference, based on plastome sequences of tRNA, rRNA and CDS regions. Tribes are indicated beside the tree. The numbers above the branches indicate bootstrap support.

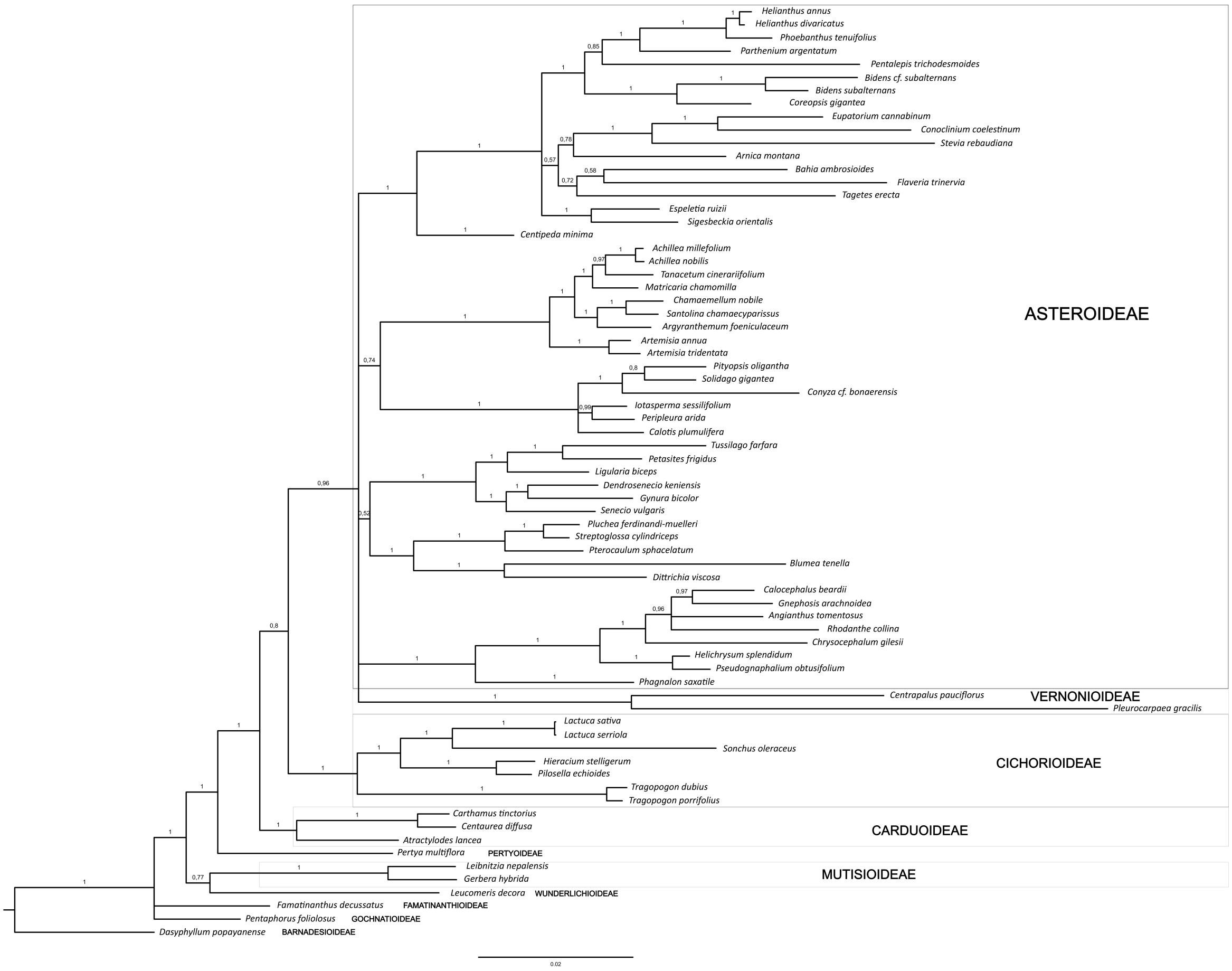

Figure S3. Phylogenetic tree of Asteraceae obtained using Bayesian inference, based on 35S rDNA sequences. Subfamilies are indicated beside the tree. The numbers above the branches indicate posterior probability.

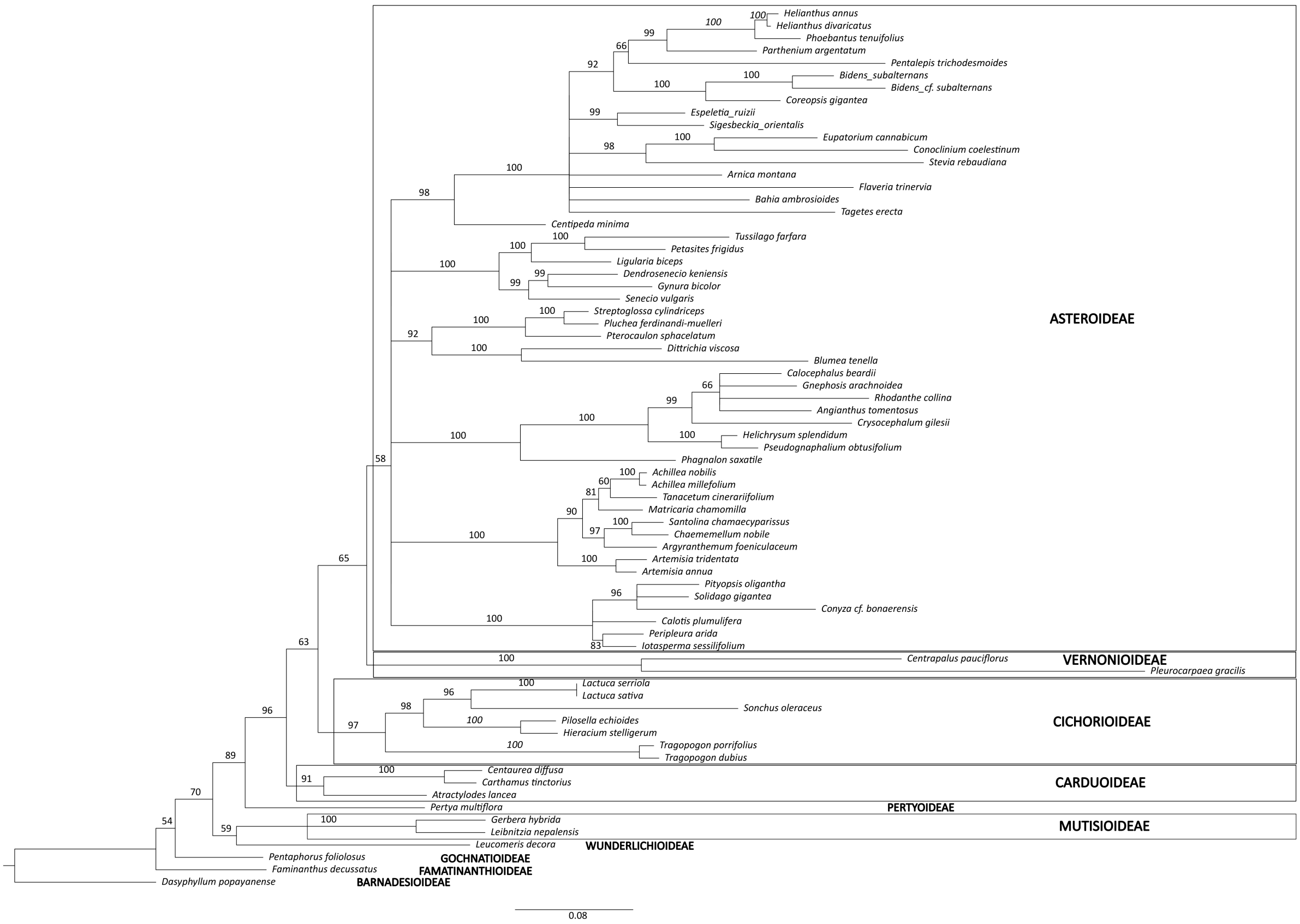

Figure S4. Phylogenetic tree of Asteraceae obtained using Maximum-likelihood inference, based on 35S rDNA sequences. Subfamilies are indicated beside the tree. The numbers above the branches indicate bootstrap support.

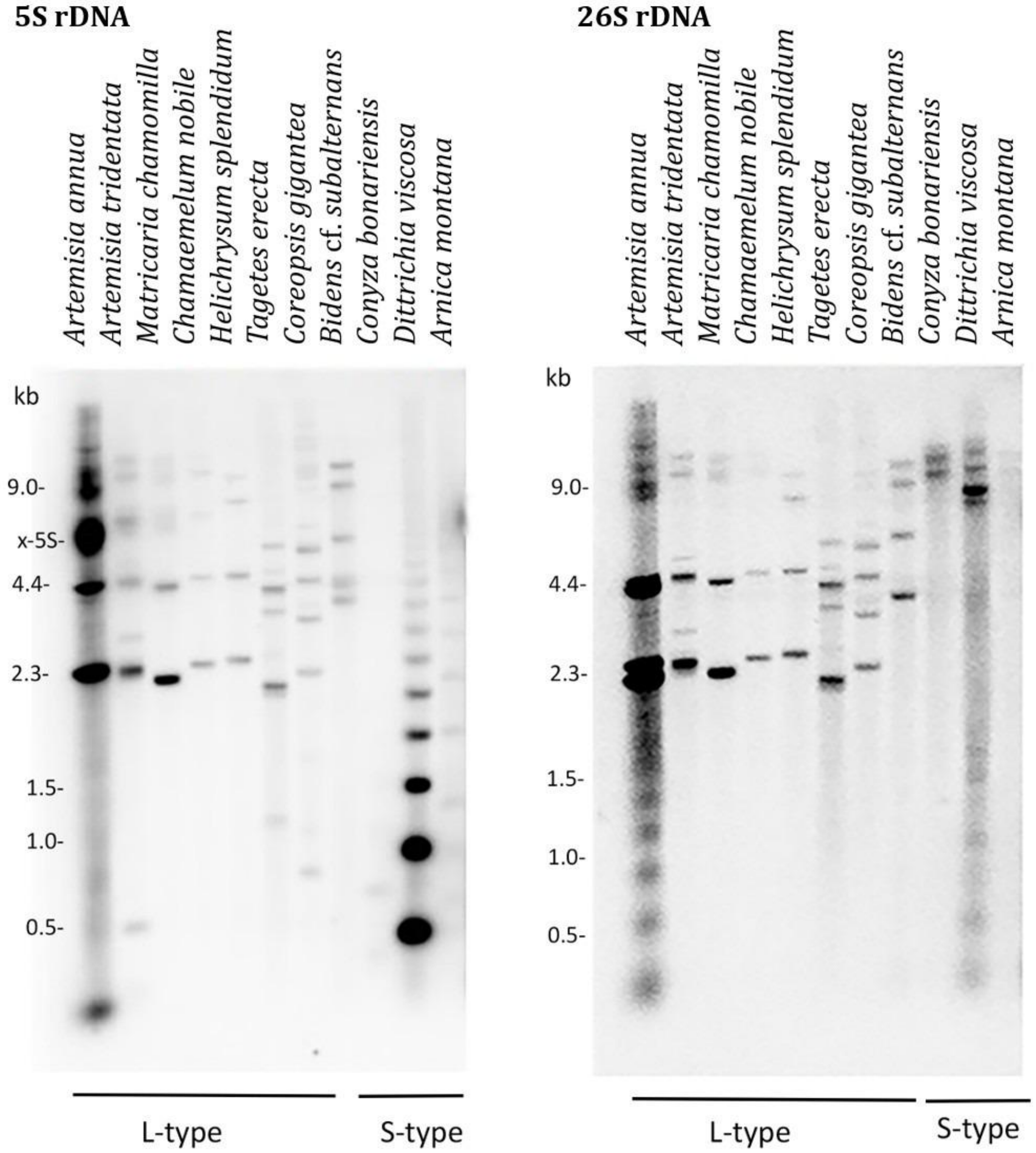

Figure S5. Southern blot hybridisation of BamHI-digested genomic DNA from selected Asteraceae species showing contrasting arrangements of 5S and 35S (26S) rDNA. Genomic DNAs were digested with BamHI, separated by agarose gel electrophoresis, transferred to membrane, and hybridised with a 5S rDNA probe (derived from an *Artemisia tridentata* 5S rDNA trimer including NTS, GenBank JX101915.1). The same membrane was then stripped and re-hybridised with a 26S rDNA probe (GenBank X13557.1). Probes were labelled with [32P]- dCTP by random-primed labelling. L-type species (bracket) show several co-migrating fragments detected by both probes, consistent with physical linkage of 5S and 35S rDNA, likely via 5S sequences embedded within the 35S intergenic spacer (IGS). S-type species (bracket) show distinct, non-overlapping hybridisation patterns: the 5S probe hybridises mainly to a ladder of low- molecular-weight fragments, whereas the 26S probe predominantly detects high-molecular-weight fragments (>9 kb), consistent with unlinked 5S and 35S arrays. In *Artemisia annua*, the x-5S band (~6 kb) denotes a prominent 5S-positive/26S-negative fragment, consistent with a 5S-containing IGS-derived fragment lacking 26S coding sequence (e.g., an IGS variant/5S insertion not spanning 26S). In *Conyza bonariensis*, the very weak 5S rDNA signal, despite detectable 26S hybridization, likely reflects limited 5S detectability due to low copy number, fragment heterogeneity and/or reduced probe affinity, rather than absence of 5S rDNA. Fragment sizes (kb) are indicated on the left.

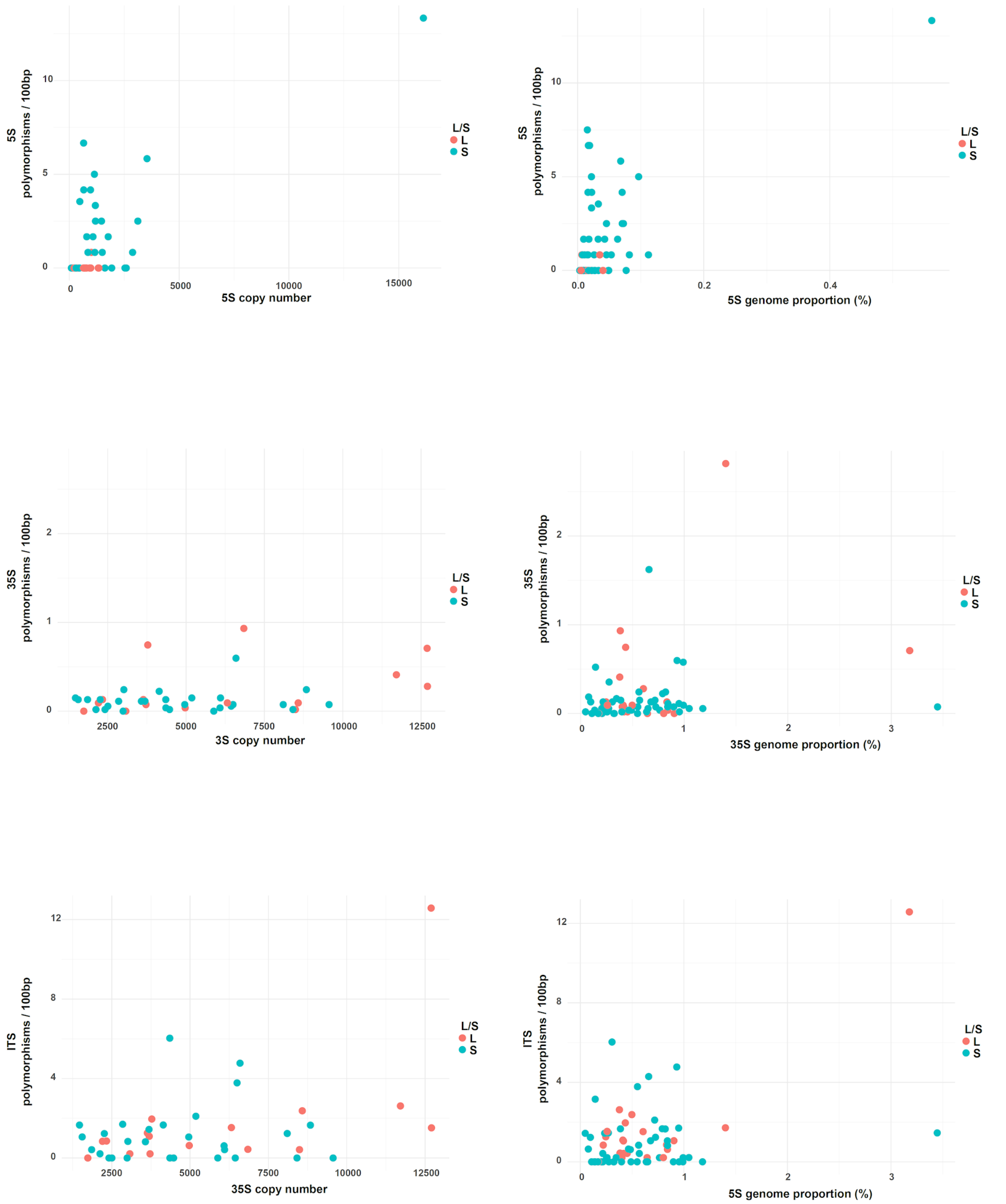

Figure S6. Scatterplots showing the relationship among intragenomic polymorphism values, copy number and genome proportion in 5S, 35S and ITS rDNA regions for species with separated arrangement (S-type, blue dots) and species showing the linked arrangement (L-type, orange dots).

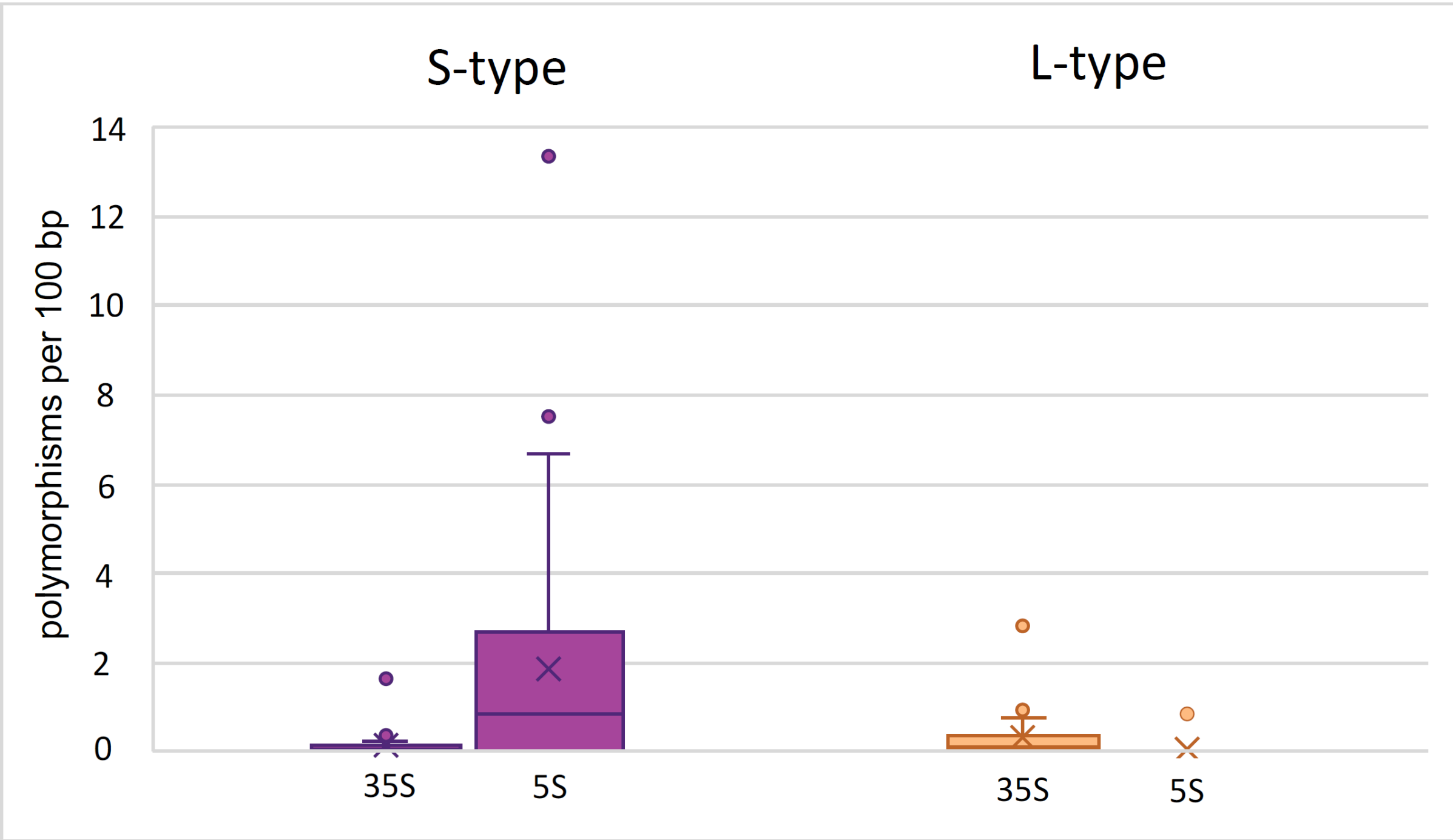

Figure S7. Boxplots of intragenomic polymorphism values in 35S (blue) and 5S (orange) rDNA regions among species showing separated arrangement (S-type) and species showing the linked arrangement (L-type).

Table S4. Collection details and herbarium information of the species sequenced on this study.

Species	Origin Region	Site / notes	Country	Collectors	Herbarium code
Achillea nobilis	Nordburgenland	E shore of Lake Neusiedl, ~6.0–6.3 km E–ESE of Frauenkirchen church	Austria	T. Barta	BCN 878581
Artemisia annua	Inner Mongolia, Bayannur province	E slopes of Helan Shan, N Temple of Helan Shan	China (PRC)	J. Vallès; S.W. Zhao	BCN 130815
Chamaemelum nobile	Baixo Alentejo, Odemira	Cavaleiro, Cabo Sardão	Portugal	A.P. Paes; P. Arsénio; J.C. Costa	BCN 879153
Conyza sp.	Catalonia, Barcelona	Caldes de Montbui	Spain	T. Garnatje	BCN 997201
Dittrichia viscosa (= *Inula viscosa*)	Catalonia, Barcelona	Caldes de Montbui	Spain	T. Garnatje	BCN 997202
Matricaria chamomilla	Commercial seeds	Cultivar Zloty Lan. SEMO a.s, 79817, Smrzice	Czech Republic	—	—
Tagetes erecta (= *T. patula*)	Commercial seeds	Cultivar Bonanza Bee. SEMO a.s, 79817, Smrzice	Czech Republic	—	—
Tragopogon dubius	Washington	Pullman (Isulate 2613-3-1)	USA	R. Matyasek	—

Table S5. Genome size estimations of Asteraceae species sequenced. Bold taxa indicates that genome size are first time estimated.

Specie	Tribe	Genome size			
		2n (pg)	SD	1C [Mbp]	1Cx [Mbp]
Achillea millefolium	Anthemideae	18.480	0.200	9036.720	2259.180
Argyranthemum foeniculaceum	Anthemideae	12.490	0.050	6107.610	6107.610
Bahia ambrosioides	Bahieae	6.450	0.020	3154.050	3154.050
Bidens subalternans	Coreopsideae	5.530	0.100	2704.170	2704.170
Conyza bonariensis	Astereae	4.220	0.040	2063.580	2063.580
Coreopsis gigantea	Coreopsideae	12.150	0.070	5941.350	5941.350
Dittrichia viscosa	Inuleae	2.300	0.020	1124.700	1124.700
Eupatorium cannabinum	Eupatorieae	4.920	0.080	2405.880	2405.880
Helichrysum splendidum	Gnaphalieae	1.670	0.020	816.630	816.630
Phagnalon saxatile	Gnaphalieae	2.228	0.011	1089.528	1089.528
Santolina chamaecyparissus	Anthemideae	10.280	0.005	5026.920	5026.920
Tussilago farfara	Senecioneae	3.520	0.041	1721.077	1721.077
